# Activation of *Arabidopsis* Seed Hair Development by Cotton Fiber-Related Genes

**DOI:** 10.1371/journal.pone.0021301

**Published:** 2011-07-11

**Authors:** Xueying Guan, Jinsuk J. Lee, Mingxiong Pang, Xiaoli Shi, David M. Stelly, Z. Jeffrey Chen

**Affiliations:** 1 Section of Molecular Cell and Developmental Biology and Institute for Cellular and Molecular Biology, The University of Texas at Austin, Austin, Texas, United States of America; 2 Department of Soil and Crop Sciences, Texas A&M University, College Station, Texas, United States of America; USDA-ARS, United States of America

## Abstract

Each cotton fiber is a single-celled seed trichome or hair, and over 20,000 fibers may develop semi-synchronously on each seed. The molecular basis for seed hair development is unknown but is likely to share many similarities with leaf trichome development in *Arabidopsis*. Leaf trichome initiation in *Arabidopsis thaliana* is activated by *GLABROUS1* (*GL1*) that is negatively regulated by *TRIPTYCHON* (*TRY*). Using laser capture microdissection and microarray analysis, we found that many putative MYB transcription factor and structural protein genes were differentially expressed in fiber and non-fiber tissues. *Gossypium hirsutum MYB2* (*GhMYB2*), a putative *GL1* homolog, and its downstream gene, *GhRDL1*, were highly expressed during fiber cell initiation. GhRDL1, a fiber-related gene with unknown function, was predominately localized around cell walls in stems, sepals, seed coats, and pollen grains. *GFP:GhRDL1* and *GhMYB2:YFP* were co-localized in the nuclei of ectopic trichomes in siliques. Overexpressing *GhRDL1* or *GhMYB2* in *A. thaliana* Columbia-0 (Col-0) activated fiber-like hair production in 4–6% of seeds and had on obvious effects on trichome development in leaves or siliques. Co-overexpressing *GhRDL1* and *GhMYB2* in *A. thaliana* Col-0 plants increased hair formation in ∼8% of seeds. Overexpressing both *GhRDL1* and *GhMYB2* in *A. thaliana* Col-0 *try* mutant plants produced seed hair in ∼10% of seeds as well as dense trichomes inside and outside siliques, suggesting synergistic effects of *GhRDL1* and *GhMYB2* with *try* on development of trichomes inside and outside of siliques and seed hair in *A. thaliana*. These data suggest that a different combination of factors is required for the full development of trichomes (hairs) in leaves, siliques, and seeds. *A. thaliana* can be developed as a model a system for discovering additional genes that control seed hair development in general and cotton fiber in particular.

## Introduction

Cotton fiber is the largest renewable source of textile materials, a sustainable alternative to petroleum-based synthetic fibers. Cotton fiber is derived from seed protodermal cells and among the premier biological systems for studying cell differentiation and development. Cotton seed hair development share many similarities with *Arabidopsis* leaf trichome development [1], which is mediated by a “trichome activation complex”. Leaf trichome initiation in *Arabidopsis thaliana* is promoted by the positive transcription regulators GLABROUS1 (GL1), TRANSPARENT TESTA GLABRA1 (TTG1), GLABRA3 (GL3), and ENHANCER of GL3 (EGL3) that are counteracted by the negative regulators TRIPTYCHON (TRY), CAPRICE (CPC), and ENHANCER of TRY and CPC1 (ETC1, 2, and 3) that encode single MYB-domain protein families [2], [3], [4], [5], [6]. GLABROUS2 (GL2) functions downstream of the GL1/TTG/GL3 complex and also plays a role in leaf trichome development [3], [7]. Some MYB factors such as AtMYB5 and AtMYB23 have minor effects on trichome initiation but regulate mucilage biosynthesis and seed coat development [8]. Moreover, trichome genes such as *TTG1* and *GL2* affect mucilage biosynthesis and columella cell formation [9], [10], suggesting a role of these trichome genes in seed coat development [11].

Several studies using cotton fiber-related genes have demonstrated a close relationship between cotton seed fibers and *Arabidopsis* leaf trichomes. *Gossypium arboreum MYB2* (*GaMYB2*) encoding a putative homolog of GL1 MYB transcription factor complements the trichomeless *gl1* mutant and induces occasional hair formation in *A. thaliana* seeds [12]. *GaHOX1*, a homeobox gene, encodes a HD-ZIP IV transcription factor, and is a functional homologue of the *A. thaliana GL2* gene [13]. Two WD-repeat genes from *Gossypium hirsutum* (*GhTTG1*) restore trichome formation in *A. thaliana ttg1* mutant plants and complement anthocyanin defects in *Matthiola incana ttg1* mutants [14]. Moreover, microrarray and gene expression analyses have uncovered many cotton fiber-related genes, including those encoding MYB transcription factors and phytohormonal regulators [15], [16]. For example, differential expression of six *MYB* genes is observed in allotetraploid cotton (*G. hirsutum* L.) [17]. Several *MYB* and *RDL* genes are expressed in fiber initials through microarray analysis [18]. GhMYB25 regulates early fiber and trichome development in cotton [19]. The data collectively suggest that *Arabidopsis* and cotton use similar transcription factors for the development of leaf trichomes and seed hairs. However, the mechanisms responsible for the differentiation of branched trichomes in vegetative tissues (leaves) and unbranched hairs in reproductive organs (seeds) may not be the same, and many seed plants including *Arabidopsis* do not produce seed hairs.

In this study, we employed microarray analysis of gene expression in fiber and ovular cells captured by laser micro-dissection and found differential expression of several hundred genes. A subset of genes, including *G. hirsutum MYB2* (*GhMYB2*), *GhMYB112b*, *GhRDL1*, and *Fb37*, were validated by quantitative RT-PCR analysis and RNA *in situ* hybridization. Furthermore, we chose *GhMYB2*, a GL1-like MYB transcription factor gene, and a downstream gene *GhRDL1* to test the hypothesis that cotton fiber-related genes can program seed hair development in *A. thaliana*. GFP:GhRDL1 and GhMYB2:YFP fusion proteins were studied in *A. thaliana* seed coat and ectopic trichomes. The functions of *GhMYB2*, a putative homolog of *GL1*, and *GhRDL1*, a putative homolog of *RD22* in *A. thaliana*, were tested by overexpressing each cotton gene alone or together in *A. thaliana* wild-type (Col-0) or *try* mutant plants. Our data revealed novel roles of cotton fiber genes in the formation of seed hairs and ectopic trichomes inside and outside of siliques in *A. thaliana*.

## Results

### Differential gene expression in cotton fiber cell initials and ovular cells captured by laser microdissection

Using the cotton spotted long oligonucleotide microarrays with probes of many fiber ESTs developed in previous studies [16], [20], [21], we studied expression of fiber-related genes in ovules, protodermal fiber cells, and fiber cell initials by laser capture microdissection (LCM) (Figure S1, A and B). Several hundred genes in each of ten comparisons were differentially expressed between fibers and non-fiber tissues (Table S1, Table S2, Figure S1C). In the comparison between inner integuments and protodermal cells (−2 and 0 DPA), fiber cell initials (2 DPA), or fibers (7 DPA) (Table S1), the number of differentially expressed genes during fiber initiation (−2, 0, and 2 DPA) was higher than that in early stages of fiber cell elongation (7 DPA) (Figure S2A). The overlap between the differentially expressed genes in different stages (−2, 0, and 2 DPA) was relatively small (2–16%) and statistically insignificant (Table S3, Figure S2A), suggesting coordinated regulation of gene expression changes during early stages of fiber development [16]. The numbers of differentially expressed genes between ovule integument versus protodermal cells (−2 DPA) or fiber cell initials (2 DPA) were statistically significantly different, suggesting that a different set of genes is expressed during ovule and fiber development (Table S4).

**Figure 1:**
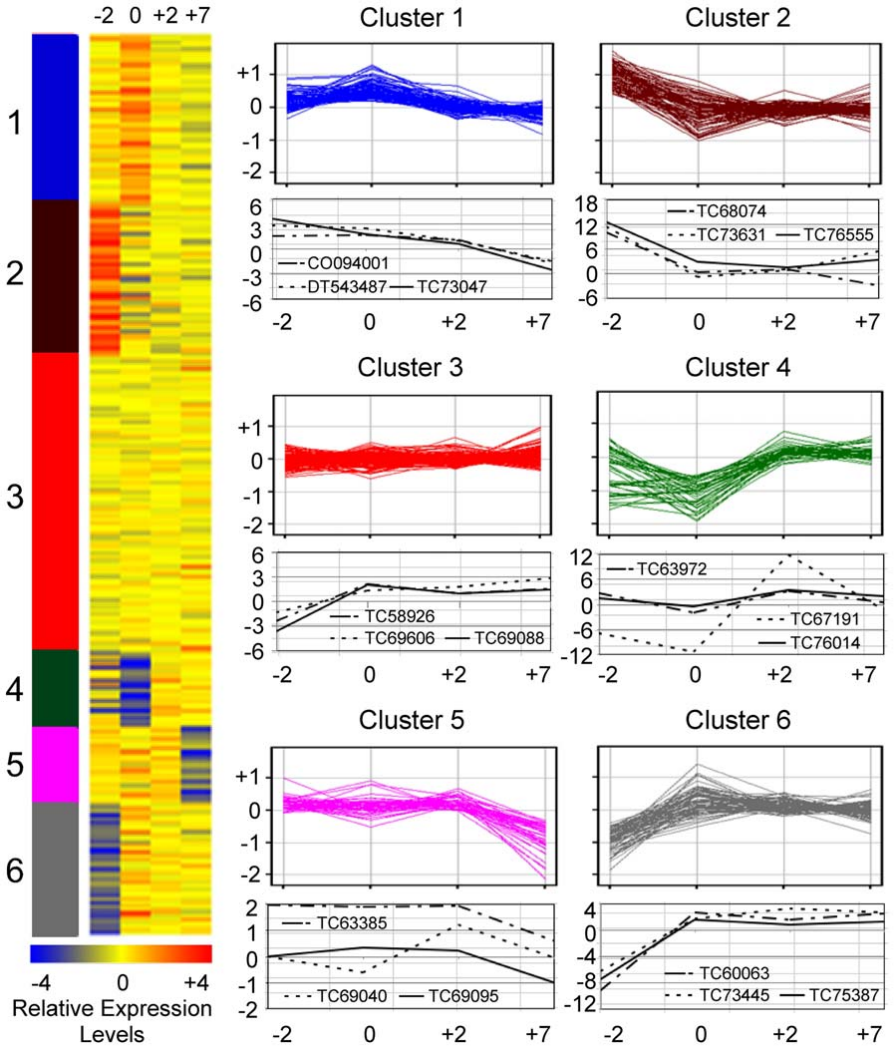
Comparative and K-means analyses of differentially expressed genes detected by microarrays. A heat map of K-means cluster analysis of differentially expressed genes in protodermal cells (−2 DPA), fiber cell initials (0 and +2 DPA), and fibers (+7 DPA). Changes of gene expression in six Gene Ontology (GO) categories are shown with the expression patterns of three representative genes shown below each cluster graph. Cluster 1: kinase activity; 2: hydrolase activity; 3: transferase activity; 4: transcription factor; 5: DNA/RNA binding activity; and 6: structural molecule. Expression of representative genes was validated by qRT-PC. ID in parenthesis is updated from ICG10.

Using gene ontology classification of molecular function, the differentially expressed genes in different stages of ovule and fiber development were clustered by K-means clustering [22]. Six groups of gene expression patterns and their representative genes were identified (Figure 1, Table S5). Expression of the genes related to transcription activity (cluster 4) decreased from −2 to 0 DPA, but increased in fiber cells initials and fibers from 2 to 7 DPA. These include genes encoding putative transcription factors (TC67191, TC75739, TC76014), phytohormone responsive factors, and a germin-like protein (TC72843), which is consistent with the previous data [21], [23]. The genes encoding putative MYB2 and MYB25 transcription factors and germin-like proteins play roles in cotton fiber cell development [12], [19]. The genes related to DNA or RNA binding activity (cluster 5) were upregulated in the protodermal cells during fiber initiation, but the expression levels decreased at 7 DPA. Two gibberellin-responsive genes (TC65219 and TC79957) and a sucrose synthase gene (*Sus*) (TC73327) belonged to this group. *SUS3* is a fiber-related protein and localized in fiber cells. Suppression of *SUS3* expression inhibits cotton fiber initiation and development [24]. The genes encoding structural molecules such as ribosomal proteins and tubulin factors (cluster 6) were highly expressed in the ovules at −2 DPA and during fiber cell development, consistent with preferential accumulation of tubulins during fiber cell elongation [23], [25] and a large proportion of ribosomes produced during rapid cell elongation [26].

### Enriched expression of *GhMYB2* and *GhRDL1* in cotton fiber

Expression patterns for a subset of genes detected by microarrays were validated by quantitative RT-PCR (qRT-PCR) analysis (Table S6, Figure S2B-G). AI730621 encoding a predicted ring zinc finger protein and TC62849 encoding a putative fiber protein 37 (Fb37) were abundantly expressed in the protodermal cells and fiber cells. The transcript levels of TC75739 (similar to *GhMYB25* and *GhMYB112b*) increased from −2 DPA to +2 DPA, while *GhMYB2* was actively expressed in young fibers (3 and 5 DPA). Transcript levels of AI072821 were high in the protodermal cells and increased in elongating fibers (7 DPA).

AI072821 is a *G. hirsutum* homolog (*GhRDL1*) of *A. thaliana* RESPONSIVE TO DESSICATION 22 (RD22)-like1. *A. thaliana* RD22 is responsive to dehydration stress and expressed in seeds [27]. A putative homolog of seed coat BURP-domain protein1 (SCB1) gene in soybean is expressed within cell walls, suggesting a role in the differentiation of the seed coat and columella cells [28]. In cotton, *G. arboreum RDL1* (*GaRDL1*) was expressed in developing fiber cells [29], and the promoter of *GaRDL1* fused with a β-glucuronidase (GUS) displayed trichome-specific expression in *A. thaliana* leaves [12]. GaMYB2 activated expression of *GaRDL1* probably through direct binding of the L1 box and MYB motif in the promoter. *GaMYB2* affects seed hair formation in *A. thaliana* [12], and *GhMYB25* affects trichome and fiber development in cotton [19], but their effects are moderate, suggesting that it may require additional downstream genes to stimulate the activities. Here we chose *GhMYB2*, a GL1-like MYB transcription factor gene, and *GhRDL1*, a gene that contains GaMYB2 binding motifs in its promoter to test the hypothesis that cotton fiber-related genes can program seed hair development in *A. thaliana*.

### Expression of *GhRDL1* in cotton fiber cell initials and seed coat in *A. thaliana* seeds

**Figure 2:**
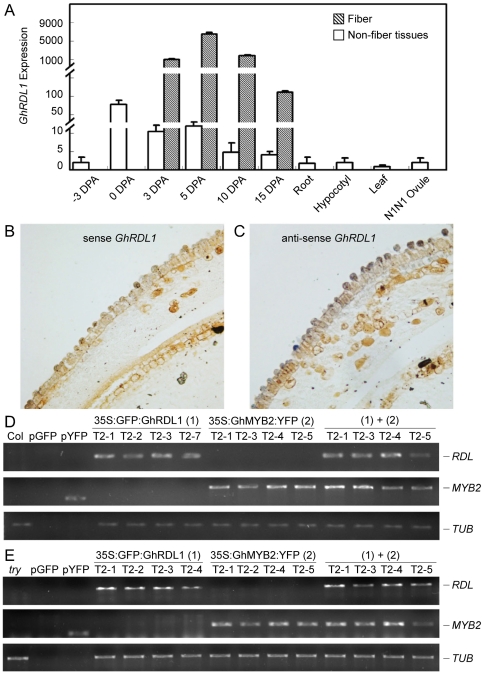
*GhRDL1* expression patterns in cotton and *GFP:GhRDL1* and *GhMYB2:YFP* transgene expression in *A. thaliana* Col and *try* mutant transgenic plants. (**A**) Quantitative RT PCR (qRT-PCR) analysis of *GhRDL1* expression in ovules, fibers, and non-fiber tissues of TM-1 and ovules (0 DPA) of N1N1. (**B–C**) RNA *in situ* hybridization in cotton ovules (0 DPA) using sense (**B**) and anti-sense (**C**) probes showing accumulation of *GhRDL1* transcripts in fiber cell initials (**C**). (**D**) RT-PCR analysis of transgene expression in *35S:GFP:GhRDL1* transgenics, *35S:GhMYB2:YFP* transgenics, and *35S:GFP:GhRDL1* and *35S:GhMYB2:YFP* double transgenics. Amplification of *Tublin* (beta 6) gene (*TUB*) was used as an RNA loading control. (**E**) RT-PCR analysis of the transgene expression as in (**D**), except that the transgenic plants were in the *try* mutant background.

The predicted protein of a cloned full-length *GhRDL1* cDNA shared 97% amino-acid sequence identity with *GaRDL1* and had a major deletion relative to *A. thaliana RD22* (*AtRD22*) (Figure S3). *GhRDL1* was expressed at higher levels in fiber cells and elongating fibers than in ovules and non-fiber tissues, including roots and hypocotyls (Figure 2A). Interestingly, *GhRDL1* expression was low in ovules of the *G. hirsutum* naked seed mutant (*N1*) that produces little or no fiber.

RNA *in situ* hybridization assays indicated that *GhRDL1* transcripts were localized specifically in fiber cell initials of cotton ovules, compared to the background signals detected using a sense RNA probe (Figure 2B). The data suggest that *GhRDL1* is a fiber-related gene in tetraploid cotton.

**Figure 3:**
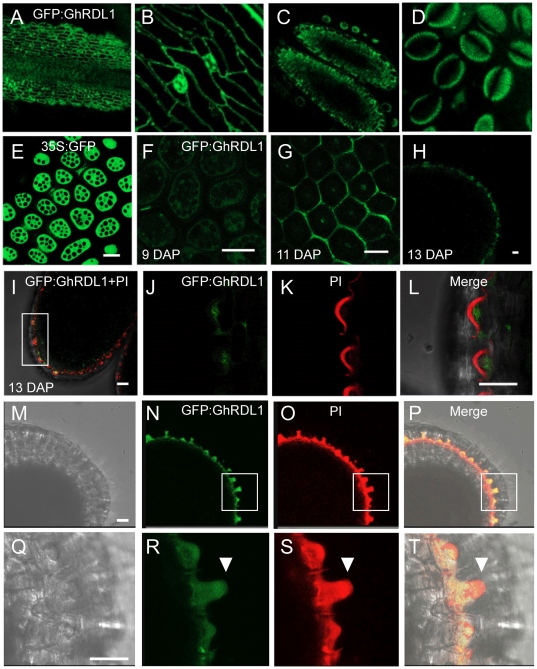
Localization of 35S:GFP:GhRDL1 in *A. thaliana* transgenic plants. (**A–D**) Localization of 35S:GFP:GhRDL1 in stem (**A**), sepal cell walls (**B**), stamen (**C**) and pollen grains (**D**). (E) 35S:GFP alone in the seed coat at 9 days after pollination (DAP). (**F–H**) Localization of 35S:GFP:GhRDL1 in cell walls of seed coat in *A. thaliana*: 35S:GFP:GhRDL1 at 9 DAP (**F**), 11 DAP (**G**), and longitudinal section of seed coat at 13 DAP (**H**). (**I**) Merged image of 35S:GFP:GhRDL1 and propidium iodide (PI, red) at 13 DAP. (**J–L**) Enlarged views of a section in (**I**) stained with GFP:GhRDL1 (**J**), PI (**K**), and merged (**L**). (**M–P**) Unstained (**M**), GFP:GhRDL1 (**N**), PI (**O**), and merged images (**P**) of *35:GFP:GhRDL* in columella cells of seed coat. (**Q–T**) Enlarged views of the selected areas (boxed in I) showing unstained (**Q**), GFP:GhRDL1 (**R**), PI (**S**), and merged (**T**) of a fiber-like cell initial (arrowheads). Scale bars represent 50 mm.

*A. thaliana* transgenic plants expressing 35S:GFP:GhRDL1 fusion protein construct were generated to test cellular localization of GhRDL1 in *A. thaliana* plants and seed coat. The transgenic plants were selected using 3∶1 segregation ratio based on antibiotic selection in T2 generation. Among ∼30 independent transgenic lines, five (*GhRDL1At1–5*) were genotyped, and four lines were used for further analysis. *GhRDL1* was uniformly expressed in four independently-derived T2 transgenic plants (Figure 2D). GFP:GhRDL1 was found around cell walls in stems, sepals, stamens, and pollen grains (Figure 3A–D). In the control plants expressing 35S:GFP, the GFP signals dispersed in the protodermal cells in all stages examined (Figure 3E). At cellular levels, the *GFP:GhRDL1* signals (green) in the *GFP:GhRDL1* transgenic plants appeared 5 days after pollination (DAP), clearly visible at 9 DAP (Figure 3F), and accumulated at high levels within cell walls of seed coat at 11 and 13 DAP, respectively (Figure 3G and 3H). The GFP:GhRDL1 signals were distributed in the epidermal cells at 13 DAP (Figure 3J). Propidium iodide (PI) is normally used to stain DNA and RNA as well as cell walls. At subcellular levels, GFP:GhRDL1 proteins (green) (Figure 3I and 3L) were localized underneath the cell walls that were stainable with PI (red) (Figure 3K and 3L), suggesting that GhRDL1 proteins accumulate in columella cells of *A. thaliana* seed coat. Indeed, localization of GhRDL1:GFP and PI aggregated at 13 DAP (Figure 3N–O), and a cell initial elongated under high magnification (arrows, Figure 3R–T). Because endogenous *RDL1* promoter had a weak activity in our hands, these data did not preclude localization of RDL1 in other cell types in addition to cell walls.

### Activation of seed hair development by overexpression of *GhRDL1*, *GhMYB2* or both

**Figure 4:**
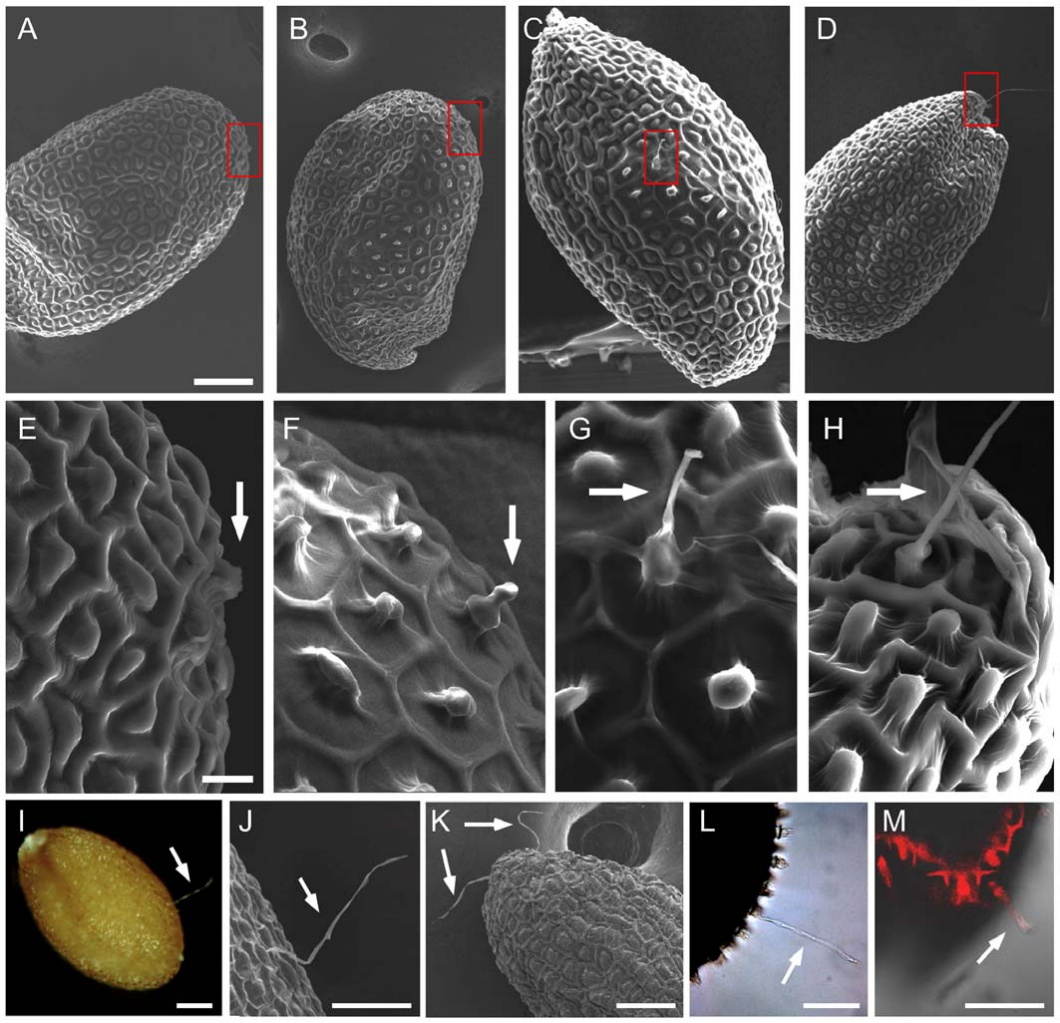
Seed hair production in the transgenic plants overexpressing *GhRDL1* alone or *GhRDL1* and *GhMYB2* together. (**A–D**) Initiation of seed hair in the *try* transgenic plants (seeds) expressing *35S:GFP:GhRDL1* alone. Scanning electron microscope (SEM) images of developing seeds at 13 days after pollination (DAP) (**A**), 15 DAP (**B**), 17 DAP (**C**), and 21 DAP (**D**). Hair-like initial was observed as early as 11 DAP. The boxed areas were zoomed in (E–H). Scale bar represents 100 µm. (**E–H**) Enlarged SEM images of corresponding boxed regions in (**A–D**). Scale bar represents 10 µm. (**I–J**) A single seed hair in a mature seed observed using light microscope (**I**) and SEM (**J**). (**K**) Multiple seed hairs (two shown) produced in a seed of the transgenic plants overexpressing both *GhRDL1* and *GhMYB2*. (**L–M**) Seed hair without staining (**L**) and PI staining (**M**) in *GhRDL1* transgenic plants. PI stained cell walls of seed coat and seed hair. Scale bars in (I–M) represent 50 µm. Arrows in (**E–M**) indicate seed hair or fiber.

Under the electron microscope (SEM), the fiber-like initial emerged at 13 DAP (Figure 4A and 4E) and 15 DAP (Figure 4B and 4F). The elongating fiber-like hair was clearly visible at 17 DAP (Figure 4C and 4D) and 21 DAP (Figure 4D and 4H). Consequently, fiber-like hairs were found in some mature seeds (Figure 4I). SEM images showed that the hairs in mature *A. thaliana* seeds were unbranched single cells (Figure 4J), similar to the fibers grown on cotton seeds [30]. The seed hair was expandable in water (Figure 4L) and stainable with PI (Figure 4M), suggesting a wall-like structure, probably similar to the secondary wall materials in cotton fiber. The seed hairs were distributed in different (chalazal, micropylar, and middle) parts of seed coat (Figure 4A–4B). Approximately ∼6% of transgenic seeds had one or two hairs (Figure 4K) with an average hair length of ∼100 µm (Table 1).

**Table 1:** Percentage and length of seed hair in *Arabidopsis thaliana* Col-0 or Col-0 *try* mutant plants overexpressing *GhRDL1*, *GhMYB2*, or both.

Line	Hairy seed (%)	Seed hair length (µm)
Col-0	0.40±0.02^a^	43.35±18.55^a^
Col-0 35S::GFP-GhRDL1	5.57±1.96^b^	93.53±11.28^b^
Col-0 35S::GhMYB2-YFP	5.88±1.78^b^	90.31±6.08^b^
Col-0 35S::GFP-GhRDL1 35S::GhMYB2_YFP	7.73±2.33^c^	113.46±25.28^c^
Col-0 *try*	5.89±1.66^b^	116.58±41.94^c^
Col-0 *try* 35S::GFP-GhRDL1	5.14±2.12^b^	115.78±8.08^c^
Col-0 *try* 35S::GhMYB2-YFP	5.95±1.45^b^	114.30±11.51^c^
Col-0 *try* 35S::GFP-GhRDL1 & 35S::GhMYB2-YFP	10.04±2.26^d^	138.91±45.08^c^

P<0.05, a, b, c and d indicate significant different groups.

**Figure 5:**
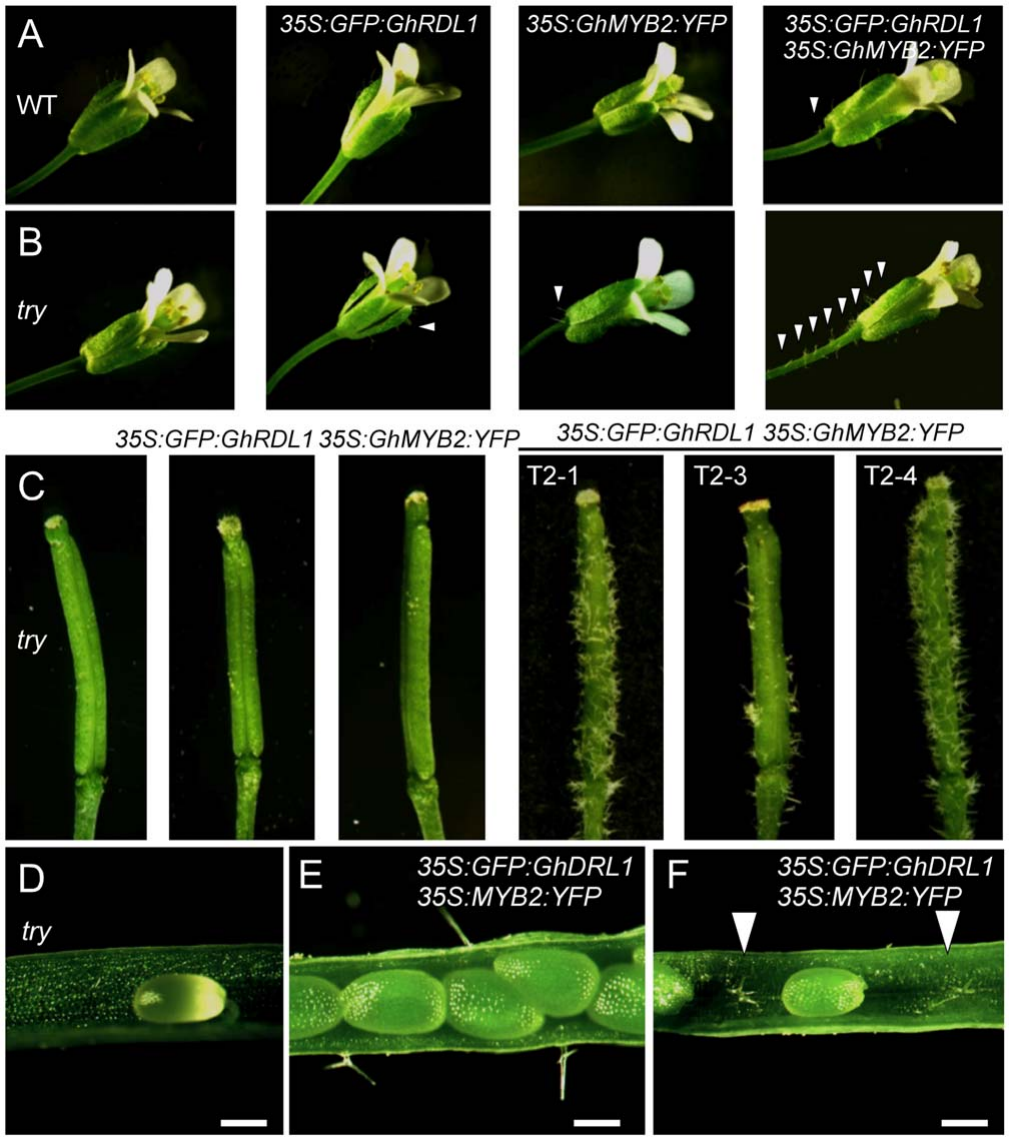
Ectopic trichome production in the transgenic plants overexpressing *GhRDL1*, *GhMYB2*, or *GhRDL1* and *GhMYB2* together. (**A**) Flowers of *A. thaliana* Col (WT, left) and transgenic plants overexpressing *GhRDL1*, *GhMYB2*, and both (right). (**B**) Flowers of *A. thaliana try* mutant (left) and transgenic plants overexpressing *GhRDL1*, *GhMYB2*, and both (right). Arrows indicate ectopic trichomes. (**C**) Ectopic trchomes outside siliques produced in three try transgenic plants (T2-1 to T2-3) overexpressing both *GhRDL1* and *GhMYB2*. (**D**) No ectopic silique trichome was observed in the *try* mutant. (**E–F**) Ectopic trichomes produced outside (**E**) and inside (**F**) siliques in the *try* transgenic plants ovexpressing *GhRDL1* and *GhMYB2*. Scale bars (**D–F**) represent 300 µm.

GaMYB2 binds the promoter of *GaRDL1* in yeast one-hybrid assays and activates *GaRDL1* expression [12]. To test if GhMYB2 and GhRDL1 act additively or synergistically to promote seed hair development, a genomic DNA fragment containing full-length *GhMYB2* was cloned into an YFP cassette. We transformed 35S:GhMYB2:YFP or 35S:GFP:GhRDL1 and 35S:GhMYB2:YFP together into Col-0. Four of ten independent T2 transgenic plants were analyzed in each event. The transgenic plants expressing *35S:GhMYB2:YFP* or *35S:GFP:GhRDL1* (Figure 2D) did not alter the distribution or density of trichomes in leaves, stems (data not shown), or pedicels (Figure 5A). In the co-transformed transgenic plants, both *GhMYB2* and *GhRDL1* were equally expressed (Figure 2D). Expressing either *GhRDL1* or *GhMYB2* in the transgenic plants produced hairs in ∼6% of seeds. In the double-gene transgenic plants, hairs were found in ∼8% of seeds (Table 1). Moreover, a few ectopic trichomes appeared in the pedicels of the double-gene transgenic plants (Figure 5A), but not in other parts of plants examined (data not shown). The data suggest additive effects of *GhMYB2* and *GhRDL1* on seed hair development in *A. thaliana*.

### Synergistic effects of *GhMYB2* and *GhRDL1* on seed hair development in the *try* mutant

These data suggest that similar to *A. thaliana GL1*, cotton *GhMYB2* and *GhRDL1* may play positive roles in trichome development, which is counterbalanced by the negative regulators such as TRY [3], [5], [31]. Constitutive expression of cotton *GaMYB2* or *GaHOX1* suppresses the leaf trichome development in *A. thaliana* [12], [13]. Removal of negative regulators such as TRY altered cell patterning of leaf trichomes and root hairs [4], [31] and might also affect seed trichome development. Indeed, *gl1*, *try*, *etc*, *gl3/egl3*, and *35S:GhRDL1* affect the formation of mucilage and columella cells. The seed surface was very rough, and some hair-like structure was observed (Figure S4). Seed hairs were found in ∼6% of seeds of *A. thaliana try* mutant plants (Table 1), suggesting a role of *try* in seed hair development. The presence of seed hair in the *try* mutants was not previously reported probably because it was not closely examined. On the contrary, seed surface in the *gl2-2* mutant was extremely smooth.

**Figure 6:**
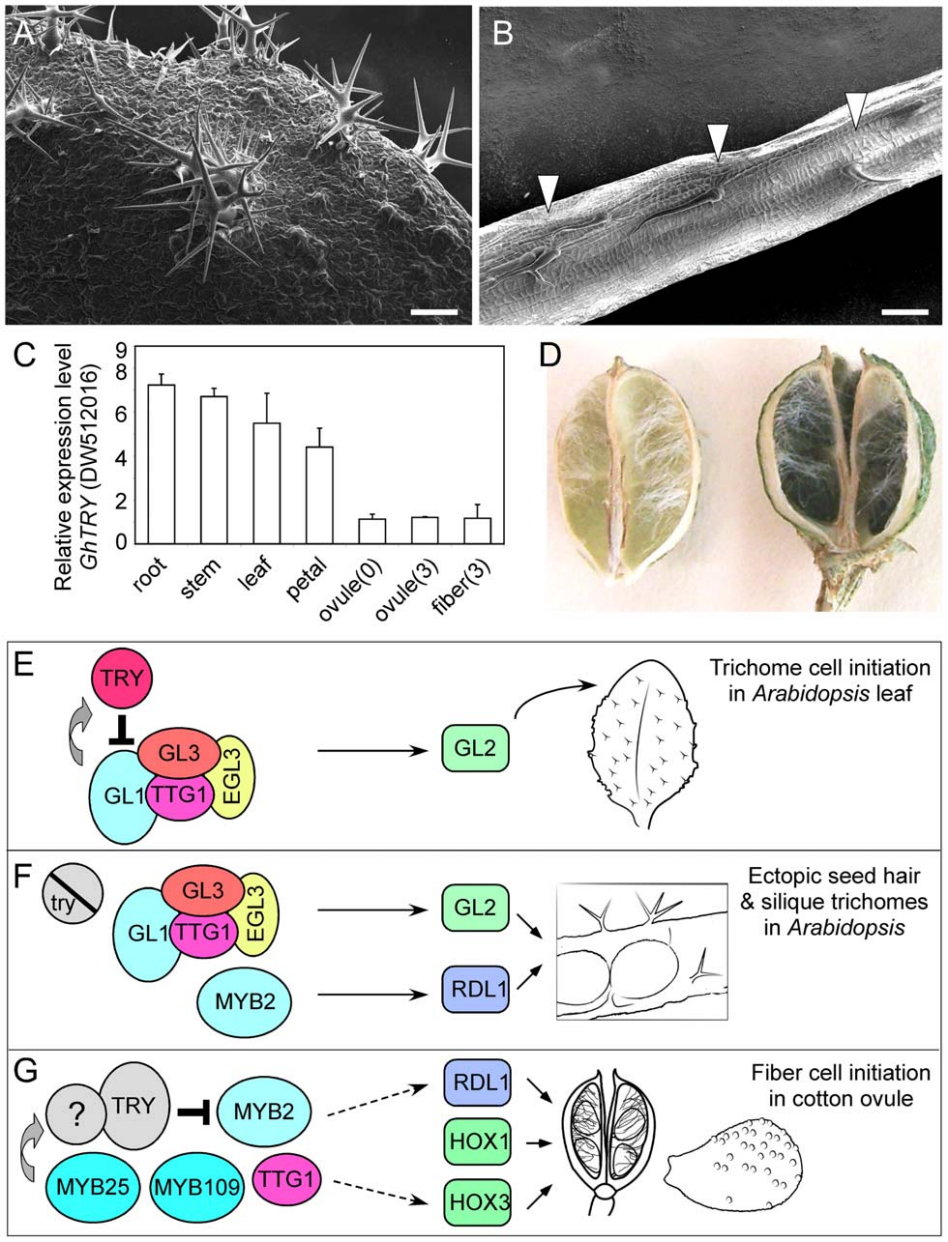
Trichome production inside siliques and cotton bolls. (**A**) An SEM image showing clustered silique trichomes in *try* transgenic plants overexpressing *GhRDL1* and *GhMYB2*. (**B**) An SEM image of branched and unbranched trichomes inside siliques of the transgenic plants. Scale bars in (A and B) represent 100 µm. (**C**) qRT-PCR analysis of a putative cotton *cpc* gene (*DW512016*) in cotton tissues (n = 3). (**D**) Unbranched trichomes (white) produced inside cotton bolls. (**E**) A model of leaf trichome development. (**F**) A model of ectopic trichome production in *Arabidopsis* silique and seed coat. (**G**) A model of seed hair production in cotton. See text for detailed explanations.

To test the effects of *GhMYB2* and *GhRDL1* on trichome and seed hair development in *try* background, *35S:GFP:GhRDL1* or *35S:GhMYB2:YFP* was transformed into *A. thaliana* Col-0 *try* mutant plants alone or together. Both *GhRDL1* and *GhMYB2* were expressed at similar levels in multiple independent transgenic plants (Figure 2E). Overexpressing *GhMYB2* or *GhRDL1* alone in the *try* mutants did not have obvious effects on trichome development in leaves or stems, except for a few ectopic trichomes developed in the pedicels and outside sepals (Figure 5B). Seed hair frequency (5–6%) in *35S:GFP:RDL1* or *35:MYB2:YFP* transgenic plants was similar to that of *try* mutant seeds (Table 1). However, expressing both *GhMYB2* and *GhRDL1* in the *try* mutant plants increased seed hair production to ∼10% of seeds and produced long seed hairs (∼120 µm) (Table 1) and multiple hairs (Figure 4K). Moreover, dense ectopic trichomes developed on sepals and pedicels (Figure 5B) as well as outside (Figure 5C and 5E) and inside (Figure 5F) siliques. The trichomes were clustered outside siliques (Figure 5C and Figure 6A). Cluster- and multi-branched trichomes are characteristic of *try* and *cpc* mutant phenotypes in the leaves but not in the siliques [3], [4], [31]. Moreover, ectopic trichomes, either branched or unbranched, were produced in the inner wall of siliques of the *try* mutant plants that expressed both *GhMYB2* and *GhRDL1* (Figure 6B). No ectopic trichomes were found on the siliques in the *try* mutants or the *try* mutants over-expressing *GhMYB2* or *GhRDL1* alone (data not shown). These data suggest that cotton fiber-related genes *GhRDL1* and *GhMYB2* act synergistically and in concert with *try* to promote trichome development in siliques and seeds in *A. thaliana*.

**Figure 7:**
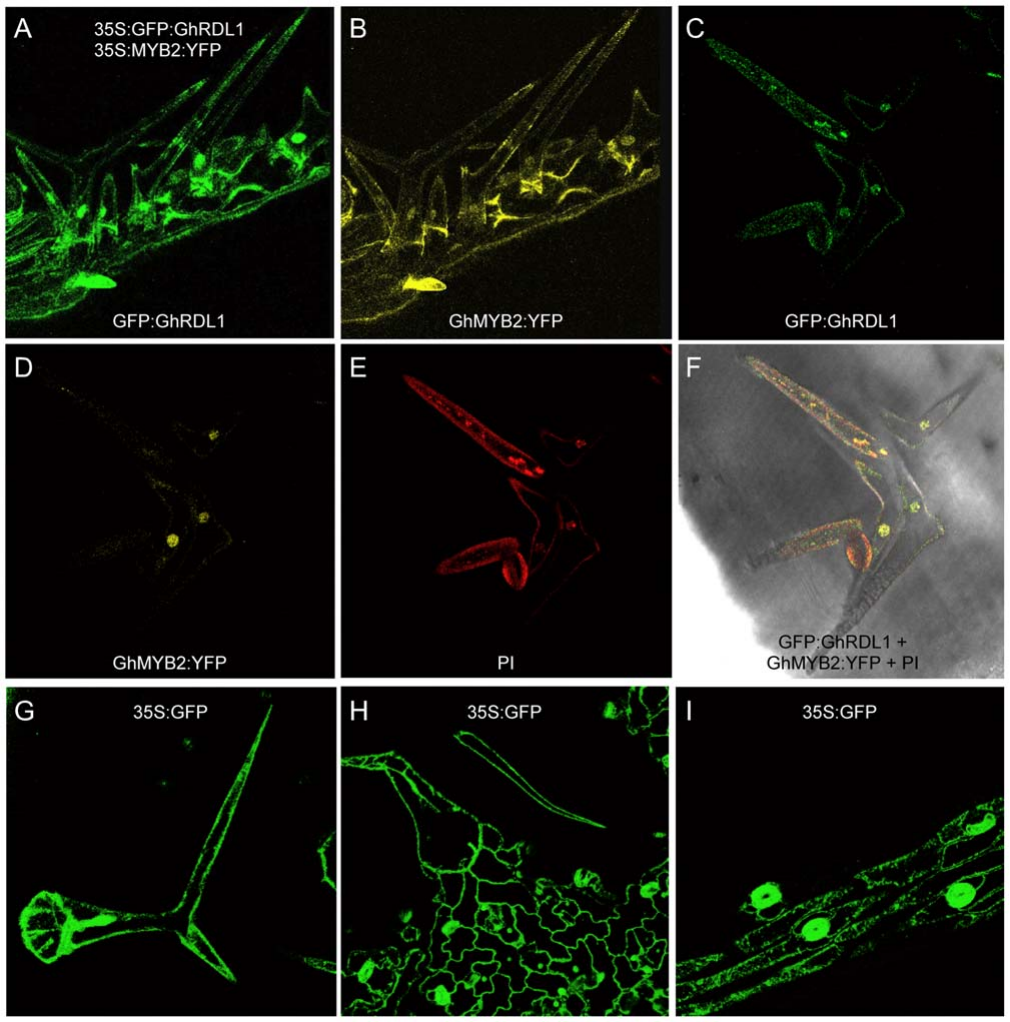
Subcellular localization of GFP:GhRDL1 and GhMYB2:YFP. (**A–B**) Localization of GFP:GhRDL1 (**A**) and YFP:GhMYB2 (**B**) in silique trichomes. (**C–F**) Silique trichomes in the *try* transgenic plants overexpressing *GhRDL1* and *GhMYB2* stained with GFP (**C**), YFP (**D**), PI (**E**, red), and merged (**F**). (**G–I**) Diffusion of GFP in leaf trichomes (**G**), epidermal cells (**H**), and siliques (**I**) in *35S:GFP* transgenic plants. GFP donuts in (**I**) indicate guard cells of stomata.

GFP:GhRDL1 was present primarily in cell walls of the silique trichomes in the *try* transgenic *A. thaliana* plants (Figure 7A). GhMYB2:YFP was primarily localized in nuclei but also in cell walls (Figure 7B). At subcellular levels, GFP:GhRDL1 was predominately localized around the cell walls and nuclei of the ectopic trichome cells in siliques (Figure 7C), whereas GhMYB2:YFP was exclusively localized in nuclei (Figure 7D). This may suggest that TRY prevents GhMYB2 from entering nuclei, and the interaction between GhMYB2:YFP and GFP:GhRDL1 promotes GFP:GhRDL1 localization in nuclei. The images of propidium iodide (Figure 7E) and GFP:GhRDL1 overlapped (Figure 7F), expect for those in the nuclei, suggesting that they are co-localized in cell walls. Co-localization of GhRDL1 and GhMYB2 in nuclei (Figure 7F) may suggest interaction of GhMYB2 with GhRDL1 in the same nuclei of the trichome cell. Whether or not GhMYB2 and GhRDL1 function in the nuclei of trichome cells remained to be tested. As controls, the staining patterns of 35S:GFP in silique trichomes (Figure 7G), epidermal cells (Figure 7H), and siliques (Figure 7I) were dispersed.

Single MYB-domain proteins such as TRY and CPC are negative regulators of GL1 and GL3 in the feedback regulation of trichome formation [3],[4]. In *A. thaliana*, a mutation in *TRY* partially suppresses the *GL1* phenotype and leads to increased numbers of epidermal and mesophyll cells and increased levels of endoreduplication in trichomes [6], [32]. A cotton EST (*DW512016*) encoding a putative GhCPC-like protein was expressed 5–8 fold higher in roots, stems, leaves, and petals than in ovules (0 and 3 DPA) or fibers (3 DPA) (Figure 6C). Different levels of putative *GhCPC* expression in fibers and roots suggest similar but different inhibitory effects of *CPC* on *de novo* patterning of trichome initiation and position-dependent cell determination during root hair development [4], [33]. Interestingly, trichomes were also present inside cotton bolls in some cotton species including *Gossypium thurberi* (Figure 6D), which is reminiscent of the ectopic trichomes developed inside siliques in the *GhMYB2* and *GhRDL1* overexpression lines in the *try* background (Figure 6B).

We proposed models to explain the development of trichomes in leaves, siliques, and seeds. In *A. thaliana*, genetic studies indicate that *GL1*, *TTG1*, *GL3*, and ENHANCER of GL3 (*EGL3*) are in the same pathway [3], [34]. Yeast two-hybrid studies showed that GL3 interacts with GL1 and TTG1. Similarly, EGL3 interacts with TTG1 and GL1 to form heterodimers with GL3 [10], [35], [36]. This complex promotes leaf trichome initiation through homeobox domain protein GL2 [4], [7] (Figure 6E). The positive effects of GL1 complex on trichome formation are counterbalanced by negative regulators TRY and CPC that are redundant but have incomplete overlapping functions [4], [5], [6], [32], [33]. When the negative regulator *TRY* is eliminated by mutation, overexpression of cotton *GhMYB2*, a putative *GL1* homologue, and *GhRDL1*, a gene expressed in the seed coat, induces regulatory perturbation of the trichome pathway, leading to the production of ectopic trichomes inside siliques and on the seed coat (Figure 6F). In cotton, several putative homologues of *GL1* genes such as MYB2 and MYB109 and MIXTA-like gene such as MYB25 are shown to affect seed fiber development [12], [19], [37] (Figure 6G). Although many factors are still missing, the interactions among MYB2, RDL1, GhTTG1, and GL2-like homeobox domain proteins such as GaHOX1 and GaHOX3 [13] promote the development of trichomes inside and outside siliques in *A. thaliana*, which may suggest the roles of these genes in cotton bolls and fibers (Figure 6D). Moreover, low expression levels of a putative *GhCPC* gene in cotton ovules and fibers is negatively correlated with cotton fiber development, similar to the negative role of *CPC* in *Arabidopsis* trichome development [3], [4].

## Discussion

### Enrichment of transcripts in several important pathways in cotton fiber development

Studying cotton gene expression during fiber cell initiation is challenging because of technical difficulties in isolating fiber initials from the whole ovules. Laser capture microdissection (LCM) has been widely used in the study of cell-type specific gene expression in plants [38], [39] including cotton [15], [40]. The most highly up-regulated genes in cotton fiber initials include those encoding proteins for the synthesis of enzymes and cell wall proteins, carbohydrates, and lipids [40]. In this study, we expanded to include ten different comparisons.

Many differentially expressed genes cannot be directly compared among different experiments. Among top 50 highly expressed genes in cotton fiber initial cells in the previous studies [15], [40], 32 (64%) were also up-regulated in the similar tissues during fiber initiation in this study. The overlapped genes include a large number of transcription factor genes, such as *GhMYB25*, *GhMYB2*, *GhMYB36*, *GhMYB38*, and *GhGL2-like*, as well as structural genes, such as cellulase synthetase gene (*CESA8*), sucrose synthase gene, fiber protein genes *Fb27* and *Fb34*, and *GhRDL1*. The identification of these MYB-related genes in multiple independent experiments suggests an important role for MYB transcription factors in the early stages of cotton fiber development [12], [19], consistent with their roles in trichome cell differentiation in *A. thaliana* [3], [6]. AtMYB106, a MIXTA-like transcription factor and homolog of cotton GhMYB25, functions as a repressor of cell outgrowth in *A. thaliana* [41]. *GaMYB2* affects seed hair formation in *A. thaliana* [12], and *GhMYB25* affects trichome and fiber development in cotton [19], but the effects are moderate, suggesting that it may require additional downstream genes to stimulate the activities. Co-regulation of a MYB transcription factor gene (*GhMYB2*) and an interacting gene *GhRDL1* whose function is unknown but highly expressed in fiber cells suggests additive effects of these two genes on fiber cell initiation. As a result, these two genes were subsequently selected for further study of their roles in parallel trichome and seed hair pathways in *A. thaliana*.

Transcriptome analysis also revealed enrichment of auxin responsive factor genes such as *ARF2* and *ARF6* and gibberellin synthetic factor transcripts in the epidermis, consistent with the requirement of gibberellin and auxin during fiber cell initiation [1], [42]. In addition, ATP binding cassette (ABC) transporter genes are involved in transporting diverse substrates including auxin across membranes [43]. Several genes encoding ABC transporters that were enriched in the fiber in this study were also abundantly expressed in a previous study [16]. Apyrase (nucleoside triphosphate-diphosphohydrolase) genes are influenced by auxin and their expression is closely related with plant growth. Expression of two *A. thaliana* apyrase (*APY1* and *APY2*) genes showed the highest expression in the tissues that accumulate high auxin levels [44]. Two putative homologs of cotton *APY* genes were low abundant during fiber initiation but highly induced during fiber elongation (at 7 DPA), indicating a role of apyrases in cotton fiber cell elongation, which was confirmed in a recent study [45].

Transcripts of the genes encoding several peroxidase and cytochrome P450 family members were enriched in elongating fibers, consistent with the enrichment of stress responsive genes during fiber development in cotton related species [46]. The biological effects of these stress responsive genes on fiber cell development remains to be tested. Comparative analysis of ten microarray datasets also supported the previous notion of temporal and spatial regulation of gene expression during fiber cell development [1], [16]. These microarray data will provide some guidance for future studies on fiber cell development in cotton and possibly trichome and seed hair development in *A. thaliana*.

### Roles of cotton fiber-related genes in *A. thaliana* seed hair development

Little is known about why some plant seeds are hairy, whereas others do not have hair at all. The gene expression and subcellular localization data suggest positive roles of *GhMYB2* and *GhRDL1* in the development of silique trichomes and seed hairs in *A. thaliana* and cotton.

*Arabidopsis* plants rarely produce seed hairs. A mutation in *TRY* induces seed hair formation. This suggests that *Arabidopsis* has the basic machinery for seed hair production, but the hair frequency is too low to be readily detected probably because other genes important to seed hair initiation are repressed, inactive or absent in the seed coat. GaMYB2 by analogy GhMYB2, a putative homolog of *A. thaliana* GL1, mediates *GaRDL1* expression through direct interaction with its promoter [12]. Similar to seed-specific expression of *RD22* in *A. thaliana* [27] and *SCB1* expression in parenchyma cells of soybean [28], GhRDL1 is predominately expressed around cell walls and columella cells of *A. thaliana* seed coat and fiber cell initials of cotton ovules, which may determine the fate of protodermal cells into seed trichome or hair. Although the function of *A. thaliana* RD22 is largely unknown, localization of cotton RDL1 in *A. thaliana* columella cells of seed coat suggest that *GhRDL1* alone or in combination with *GhMYB2* increases a potential of transforming protodermal cells into seed hairs. A soybean homolog of AtRD22 is a seed coat BURP-domain protein1 (SCB1) and is expressed within cell walls [28]. GhRDL1 and AtRD22 share 63% amino acid sequence similarities in the BURP domain. AtRD22 is a seed dehydration responsive protein and is inducible by abscisic acid [27]. High levels of *GhRDL1* expression during cotton fiber initiation and elongation suggest a dehydration-like condition during fiber cell development, probably resulting from rapid accumulation of glucose and the other secondary metabolites.

Notably, temporal regulation of *RDL1* is different in *A. thaliana* seed coat and cotton ovules. *GFP:GhRDL1* was expressed in *A. thaliana* ovules 5 days after pollination, whereas *GhRDL1* expression commenced immediately on the day of fiber initiation (0 DPA) and peaked during early stages of fiber elongation (5–7 DPA), suggesting a role of *GhRDL1* in both fiber cell initiation and elongation. The time delay is probably related to the absence of some factors in protodermal cells in the seed coat of *A. thaliana*, and accumulation of GhMYB2 and GhRDL1 in the absence of *try* has pushed columella cells to form elongating fibers. Hair-like spikes in *try* and *etc* mutants and *35S:GhRDL1* lines were evenly distributed on the seed surface (Figure S4), suggesting that all seeds are affected, and many cells are potentiated to become fiber cells. It is conceivable that additional genes are required to fully activate the regulatory network of *A. thaliana* trichome development [47], [48] and seed hair production. Seed hair development in *A. thaliana* may be similar to fiber development in cotton, which requires two major steps: fiber cell initiation and fiber cell elongation. Like trichome development, initiation of a seed hair cell requires positive regulators such as GL1 or GhMYB2. Elongation of seed hair in *A. thaliana* results from the elongation of columella cells, which consists of cell wall materials. This is reminiscent of primarily secondary cell wall formation in the late stage of fiber cell elongation in cotton [30], [49]. The current *A. thaliana* lines may not have fiber elongation factors. As a result, the seed hair may be very short (∼100 µm), and many short hairs are lost during sample preparation or simply invisible using regular optical microscope. The *A. thaliana try* transgenic plants overexpressing *GhMYB2* and *GhRDL1* can be used to screen for additional cotton fiber genes during seed hair development. This will provide a novel genetic system for the discovery and functional analysis of the cotton genes that fully activate seed fiber development in *A. thaliana*, cotton and other plants.

## Methods

### Plant materials

*Gossypium hirsutum* L. cv. TM-1, isogenic naked seed mutant (N1N1) (>6 generations of selfing) and *Gossypium thurberi* were grown in a greenhouse. Flowers were tagged on the day of anthesis, and ovules were harvested at 0 and 2 DPA. Fibers (7 DPA) were dissected from the cotton bolls. For each genotype, three biological pools each with ten plants were grown. Ovules or fibers were dissected, and the fresh tissues were frozen in liquid nitrogen and stored in a −70°C freezer and subjected to RNA extraction.

Seeds of *Arabidopsis thaliana* Columbia-0 (Col-0), *try*, *gl1*, *try*, *etc*, *gl3/egl3* (all in Col-0 background) were sterilized with 10% (v/v) bleach for 10 min, followed by two washes in sterile water. Seedlings were germinated on germination medium, and seedlings were transferred to soil (MetroMix 200, Sungro, Bellevue, WA) and grown in 16 h photoperiod at 22°C. T-DNA insertion mutant of *try* (SALK_029760) was obtained from *Arabidopsis* Biological Resource Center.

### EST selection and 70-mer oligonucleotide design for microarrays

The cotton oligonucleotide microarray was designed from several cotton EST libraries, which included three sets (1,154, 12,006, and 9,629) of oligonucleotide probes, making a total 22,789 of oligonucleotides on a single chip [20]. The two sets were derived from enriched ESTs in ovules and fibers [21] or oligonucleotides with known function genes [16].

### Laser capture microdissection (LCM)

Cotton ovules collected at −2, 0, and 2 DPA were subjected to tissue fixation [40]. The fixed ovules were embedded using Tissue-Tek® Optimal Cutting Temperature in cryo-mold (Sakura Finetek U.S.A., Torrance, CA). The embedded ovules were frozen immediately in liquid nitrogen and stored at −80°C.

Cryosectioning was performed using a Leica Cryostat (Leica Microsystems, Bannockburn, IL). The block was equilibrated at −20°C for 1 hour and cryosectioned at 10 µm. The slides with cryosectioned ovules were dehydrated in a series of ethanol (70%, 95%, and 100%) for 2 min each on ice and transferred to histoclear (National Diagnostics, Atlanta, GA).

Individual epidermis (−2 DPA) or fiber cell initials (2 DPA) were catapulted (Figure S1B) using a PALM laser capture system (P.A.L.M. Microlaser Technologies AG Inc., Bernried, Germany), and approximately 1,000 cells were collected into a tube containing 45 µl of RNALater (Ambion, Austin, TX) for RNA extraction.

### Microarray experimental design

All data is MIAME compliant and that the raw data has been deposited in a MIAME compliant database (E.g. ArrayExpress, GEO), as detailed on the MGED Society website http://www.mged.org/Workgroups/MIAME/miame.html. The microarray data are under the series accession number GSE17378.

A combination of loop and reference designs was used for microarray analysis of gene expression changes in four developmental stages (Figure S1). LCM was used to separate epidermis (−2 DPA) and fiber cell initials (0 DPA and 2 DPA) form ovules at −2 DPA, 0 DPA, and 2 DPA. Fibers were also manually dissected from ovules at 7 DPA. One loop included gene expression comparisons between epidermis (−2 DPA), fiber cell initials (0 DPA), ovules (0 DPA), and ovules (−2 DPA), while the other loop was used to compare gene expression changes between fiber cell initials at 0 and 2 DPA and ovules at 0 and 2 DPA. Gene expression changes were also compared between fiber cell initials at 0DPA and fibers at 7 DPA and between the ovules at 0 and 7 DPA.

### Microarray hybridization and statistical analysis

Captured cells were homogenized in 500 µl of RNA extraction buffer for RNA extraction [21]. Total RNA was amplified using an Amino Allyl MessageAmp™ aRNA amplification Kit (Ambion, Austin, TX) in two rounds of amplification. Amplified RNA was coupled with Cy3 or Cy5 dyes (Amersham Biosciences, Piscataway, NJ) and purified using Qiagen RNeasy mini column (Qiagen, Germantown, MD). About 1 µg of fragmented Cy3- and Cy5-labeled aRNA probes was used for hybridization. In each comparison, two dye-swaps with two biological replications were performed, resulting in a total of eight slide hybridizations. Hybridization and washing were performed as previously described [16].

Microarray data were analyzed as previously described [50]. A standard t-test statistics was used for this comparison, based on the normality assumption for the residuals. A false discovery rate (FDR) of a = 0.05 [51] was employed to control the family-wise error rate. The genes with a significant p value were further analyzed by fold-expression changes. K-means clustering using Euclidean distance measurement [22] was employed to compare differentially expressed genes across different developmental stages and in different tissue types. Wilcoxon rank-sum test [52] was performed to examine how each set of differentially expressed genes is different from each other (Table S3).

### RT-PCR and quantitative RT-PCR (qRT-PCR) analyses

qRT-PCR reaction was carried out in a final volume of 20 µl containing 10 µl SYBR Green PCR master mix (Applied Biosystems, Foster City, CA), 1 µM forward and reverse primers (Table S6), and 0.1 µM cDNA template in a ABI7500 Real-Time PCR system (Applied Biosystems, Foster City, CA). Cotton *HISTONE H3* (AF024716) was used to normalize the amount of RT-PCR products [12]. All reactions were performed in three replications using a dissociation curve as a control for the primer dimers.

Semi-quantitative RT-PCR was used to detect the expression levels of *GhRDL1* and *GhMYB2* in single-gene and double-gene transgenic plants using the primer pairs shown in Table S6. Expression of *TUB* (AT5G12250, BETA-6 tublin) was used as control.

### Cloning, transformation, and transgenic plants

A full-length cDNA of *GhRDL1* (AY072821) was amplified in the epidermis of 0 DPA ovules using a gene specific primer pair (Table S6). The amplified fragment was cloned into pGEM T-easy vector (Promega, Madison, WI) and sequenced. The *GhRDL1* insert was subcloned into *Bam*HI and *Not*I-digested 35SpBARN plant expression vector and named 35S:GhRDL1. For GFP and YFP fusion constructs, the cDNA of *GhRDL1* and gDNA of *GhMYB2* were amplified using primer pairs (Table S6) and cloned into pCGTBG (DQ370423.1) and pEarleyGate101, respectively.

*Agrobacterium tumefaciens* strain GV3101was used for floral dip transformation in *A. thaliana* [53]. pBARN and pCsGFPBT (Genbank DQ370426, 35S:GFP) vectors alone were transformed as controls. The transgenic plants were named 35S:GhRDL1, 35S:GFP:GhRDL1, 35S:GhMYB2:YFP, and 35S:pBARN, respectively. The transformed seedlings were genotyped using the primer pairs provided in Table S6.

Hygromycin (25 ml/L) and BASTA (40 ml/L) were used for selecting transgenic plants expressing pCGTBG_GhRDL1 (35S:GFP:GhRDL1) and pEarleyGate101_GhMYB2 (35S:GhMYB2:YFP), respectively.

Co-transformation using pCGTBG_GhRDL1 and pEarleyGate101_GhMYB2 was used to generate transgenic plants expressing both *GFP:GhRDL1* and *GhMYB2:YFP*. The *Agrobacterium* GV3101 cells containing pCGTBG_GhRDL1 and pEarleyGate101_GhMYB2, respectively, were grown in 250 ml liquid media until OD600>1.8. The cells were harvested by centrifugation and resuspended with 500 ml sucrose buffer containing a few drops of silwet L77. A mixture containing 250 ml each of GV3101_pCGTBG_GhRDL1 and GV3101_pEarleyGate101_GhMYB2 culture was used for transformation. T0 seeds were germinated on media plate containing 25 ml/L Hygromycin and 40 ml/L BASTA. A total of 30–40 positive transgenic lines were used for further analysis.

### Microscopy and histology

Scanning electronic microscopy (SEM) was performed using a modified protocol [54], [55]. The samples were scanned and analyzed using a Zeiss Supra 40 VP SEM with an accelerating voltage of 5 kV and a working distance of 39 mm. Images were scanned and stored as TIFF files.

The fluorescent samples were examined using a Leica SP2 AOBS Confocal microscope. The PALM laser-capture system, SEM, and confocal microscope are located in the Microscopy and Imaging Facility at The University of Texas at Austin.

### RNA *in situ* hybridization

A *GhRDL1* cDNAs fragment was amplified by PCR using the primer pair (GhRDL1-probe_F: CCAAACTAGGGAAAGTTGAT; GhRDL1-probe_R: TTACTTAGGGACCCAAACAA) and cloned into pGEM-T (Promega). Digoxigenin-labeled sense and antisense probes were synthesized with T7 or SP6 RNA polymerase (Roche, Indianapolis, IN, USA). *G. hirsutum* L. cv. TM-1 ovules were collected at 0 DPA and fixed in formalin/acetic acid/ethanol (1∶1∶18). Paraffin-embedded ovules were sectioned to 8 µm thickness for RNA *in situ* hybridization and detection using a published protocol [12].

## Supporting Information

Table S1 **List of differentially expressed genes.**
(XLS)

Table S2 **Number of differentially expressed genes.**
(DOC)

Table S3 **Wilcoxon rank sum test of differentially expressed genes.**
(DOC)

Table S4 **A subset of top differentially expressed genes in the epidermis or fibers.**
(DOC)

Table S5 **A list of representative genes in each cluster.**
(DOC)

Table S6 **Primer sequences used for RT-PCR and quantitative RT-PCR analyses.**
(DOC)

Figure S1 **Microarray experimental design and application of laser capture microdissection (LCM).** (**A**) Development of cotton fiber. (**B**) Application of LCM in fibers (2 DPA, upper panel) and inner integuments of ovules (2 DPA, lower panel). The captured cells were removed (right photos in each panel). (**C**) Microarray experimental design. Arrows denote dye swaps used in each hybridization (red: Cy5, green: Cy3).
(TIF)

Figure S2 **qRT-PCR validation of six differentially expressed genes detected by microarrays.** Gene expression was analyzed in ten tissues, namely, leaves (L), petals (P), protodermal cells (−2E), fiber cell initials (0E, 0 DPA and 2E, 2 DPA), fibers (7F, 7 DPA), ovules at −2 DPA (−2O), 0 DPA (0O), 2 DPA (2O), and 7 DPA (7O), respectively. (**A**) Ring zinc finger protein (AI730621). (**B**) Fiber protein 37 (TC62849). (**C**) Transcription factor (TC74600). (**D**) *GhMYB112b* (TC75739). (**E**) *GhRDL* (AY072821). (**F**) ABC transporter (TC75912).
(TIF)

Figure S3 **Sequence alignment of REPOSNSE TO DESSICATION 22 (RD22) and RD22-like (** ***RDL*** **) genes.** GenBank accession numbers are AAL67991 (*Gossypium hirsutum* RDL1, GhRDL1), AAT66912 (*Gossypium arboreum* RDL1, GaRDL), and Q08298 (*Arabidopsis thaliana* RD22, AtRD22).
(DOC)

Figure S4 **Effects of trichome regulatory genes on mucilage formation and columella cell morphology.** Col-0: *A. thaliana* Col-0 shows relatively smooth seed surface. Hair-like structure was observed in *try*, *etc*, *35S:GFP:GhRDL1* and *35S:GFP:GhRDL1x35S:AtGL2* seeds. The seed surface was rough, but no obvious hair-like structure was observed in *gl1* and *gl3/egl3* seeds. The seed surface of *gl2* seed was extremely smooth, suggesting loss of mucilage and columella cells.
(TIF)
