# Supplementary material for: Activation of Arabidopsis Seed Hair Development by Cotton Fiber-Related Genes
Source: PLoS One. 2011 Jul 11;6(7):e21301. doi: 10.1371/journal.pone.0021301 (PMC3136922; doi:10.1371/journal.pone.0021301)
Supplement: Table S2 — Number of differentially expressed genes. (DOC) [file pone.0021301.s002.doc]

**Table S2. Number of differentially expressed genes**

| Experiment | Comparison | No. of upregulated genes | No. of down-regulated genes |
| --- | --- | --- | --- |
| 1 | -2 DPA (E) vs. -2 DPA (O) | 622 | 430 |
| 2 | 0 DPA (E) vs. 0 DPA (O) | 972 | 742 |
| 3 | 2 DPA (FC) vs. 2 DPA (O) | 668 | 268 |
| 4 | 7 DPA (F) vs. 7 DPA (O) | 432 | 428 |
| 5 | 0 DPA (E) vs. -2 DPA (E) | 371 | 237 |
| 6 | 0 DPA (E) vs. 2 DPA (FC) | 53 | 412 |
| 7 | 0 DPA (E) vs. 7 DPA (F) | 1207 | 710 |
| 8 | 0 DPA (O) vs. -2 DPA (E) | 794 | 195 |
| 9 | 0 DPA (O) vs. 2 DPA (O) | 104 | 293 |
| 10 | 0 DPA (O) vs. 7 DPA (O) | 255 | 603 |
| E: protodermal cells; O: inner ovules; FC: fiber cells initials; F: fibers; Up- and down-regulation is relative to the expression levels in E, FC, or F (for experiment 1-4) or in E or O at 0 DPA (for experiments 5-10). | | | |
