# Supplementary material for: Activation of Arabidopsis Seed Hair Development by Cotton Fiber-Related Genes
Source: PLoS One. 2011 Jul 11;6(7):e21301. doi: 10.1371/journal.pone.0021301 (PMC3136922; doi:10.1371/journal.pone.0021301)
Supplement: Table S3 — Wilcoxon rank sum test of differentially expressed genes. (DOC) [file pone.0021301.s003.doc]

**Table S3. Wilcoxon rank sum test^a^ of differentially expressed genes**

| Comparison | Test | Hypothesis | P value | Conclusion^c^ |
| --- | --- | --- | --- | --- |
| Epidermis vs. inner ovules | 1 | Ho: µ1^b^(-2 DPA) = µ2 (0 DPA)  Ha: µ1 (-2 DPA) ≠ µ2 (0 DPA) | 0.002 | cannot reject Ho  µ1 and µ2 are similar. |
|  | 2 | Ho: µ2 (0 DPA) = µ3 (2 DPA)  Ha: µ2 (0 DPA) ≠ µ3 (2 DPA) | 0.701 | cannot reject Ho  µ2 and µ3 are similar. |
|  | 3 | Ho: µ3 (2 DPA) = µ1 (-2 DPA)  Ha: µ3 (2 DPA) ≠ µ1 (-2 DPA) | 0.000 | can reject Ho  µ3 and µ1 are different. |
| Epidermis | 1 | Ho: µ5 (-2 DPA) = µ6 (2 DPA)  Ha: µ5 (-2 DPA) ≠ µ6 (2 DPA) | 0.676 | cannot reject Ho  µ5 and µ6 are similar. |
|  | 2 | Ho: µ6 (2 DPA) = µ7 (7 DPA)  Ha: µ6 (2 DPA) ≠ µ7 (7 DPA) | 0.742 | cannot reject Ho  µ6 and µ7 are similar. |
|  | 3 | Ho: µ7 (-2 DPA) = µ5 (7 DPA)  Ha: µ7 (-2 DPA) ≠ µ5 (7 DPA) | 0.895 | cannot reject Ho  µ7 and µ5 are similar. |
| Inner ovules | 1 | Ho: µ8 (-2 DPA) = µ9 (2 DPA)  Ha: µ8 (-2 DPA) ≠ µ9 (2 DPA) | 0.040 | cannot reject Ho  µ8 and µ9 are similar. |
|  | 2 | Ho: µ9 (2 DPA) = µ10 (7 DPA)  Ha: µ9 (2 DPA) ≠ µ10 (7 DPA) | 0.000 | can reject Ho  µ9 and µ10 are different. |
|  | 3 | Ho: µ10 (-2 DPA) = µ8 (7 DPA)  Ha: µ10 (-2 DPA) ≠ µ8 (7 DPA) | 0.015 | cannot reject Ho  µ10 and µ8 are similar. |
| ^a^Tests were performed with differentially expressed genes (400 of up-regulated genes and down-regulated genes, total 800 genes) in each experiment.  ^b^The number indicates the experiment number in Table 3.1.  ^c^The conclusions were drawn based on the p value of 0.001. | | | | |
