# Supplementary material for: Activation of Arabidopsis Seed Hair Development by Cotton Fiber-Related Genes
Source: PLoS One. 2011 Jul 11;6(7):e21301. doi: 10.1371/journal.pone.0021301 (PMC3136922; doi:10.1371/journal.pone.0021301)
Supplement: Table S4 — A subset of top differentially expressed genes in the epidermis or fibers. (DOC) [file pone.0021301.s004.doc]

**Table S4. A subset of top differentially expressed genes in the epidermis or fibers**

| Developmental stages | EST ID | CGI10 ID | Tentative annotation | Fold-change  (Epidermis or fiber cells/inner layer) |
| --- | --- | --- | --- | --- |
| -2 DPA | TC67395 | TC188208 | Putative uncharacterized protein | 4.72 |
|  | TC75139 | TC228904 | Putative uncharacterized protein | 4.12 |
|  | TC64801 | TC201122 | Putative uncharacterized protein | 4.09 |
|  | TC76416 | TC185929 | S-adenosylmethionine synthetase | 4.00 |
|  | TC69935 | TC186709 | Putative uncharacterized protein | 3.88 |
|  | TC70372 | TC180410 | Probable protein arginine N-methyltransferase 6 | 3.88 |
|  | TC69404 | TC222540 | Putative uncharacterized protein | 3.79 |
|  | TC68099 | TC184426 | Putative uncharacterized protein | 3.73 |
|  | TC69145 | TC193602 | Putative uncharacterized protein | 3.66 |
|  | TC65120 | TC161274 | Putative uncharacterized protein | 3.66 |
|  | TC73481 | TC222022 | S-adenosylmethionine decarboxylase | 3.62 |
|  | TC68602 | DW501541 | Putative uncharacterized protein | 3.61 |
|  | TC66854 | TC212604 | Putative uncharacterized protein | 3.59 |
|  | TC79925 | TC189873 | Expressed protein | 3.59 |
|  | TC78618 | TC202643 | Putative uncharacterized protein | 3.59 |
|  | TC70554 | TC224220 | Putative uncharacterized protein | 3.58 |
|  | TC69404 | BF272679 | Putative uncharacterized protein | 3.56 |
|  | TC79741 | no hit | no data available | 3.55 |
|  | TC75415 | TC185959 | Putative uncharacterized protein | 3.54 |
|  | TC72823 | TC206222 | Putative uncharacterized protein | 3.53 |
|  | DT464263 | DT464263 | Putative uncharacterized protein | 3.53 |
|  | TC59840 | DW513212 | LIM domain protein GLIM1a | 3.37 |
|  | DT456520 | DT456520 | *Gossypium hirsutum* cDNA clone GH_ON16H01 5' | 3.37 |
|  | TC73260 | [BG445910](http://www.ncbi.nlm.nih.gov/entrez/query.fcgi?cmd=search&doptcmdl=genbank&db=nucleotide&term=BG445910) | C4-dicarboxylate transporter small subunit | 3.34 |
|  | TC71873 | TC155278 | Putative uncharacterized protein | 3.34 |
|  | BQ412677 | BQ412677 | Putative uncharacterized protein | 3.34 |
|  | TC68149 | DW502449 | Ethylene response factor 1 | 3.34 |
|  | TC69107 | TC213728 | Putative uncharacterized protein | 3.32 |
|  | TC77131 | TC209801 | Putative uncharacterized protein | 3.29 |
|  | CO108709 | CO108709 | Putative plasma membrane ATPase | 3.28 |
|  | TC77127 | TC206916 | Putative uncharacterized protein | 3.26 |
|  | TC78190 | TC206958 | Putative uncharacterized protein | 3.25 |
|  | TC60437 | TC181772 | Adenine phosphoribosyltransferase | 3.24 |
|  | DT553858 | TC210483 | Putative uncharacterized protein | 3.21 |
|  | BQ413377 | BQ413377 | Beta 1 3-glycosyltransferase-like protein I | 3.21 |
|  | TC60925 | CO071805 | Putative uncharacterized protein | 3.15 |
|  | TC70595 | ES832986 | Putative uncharacterized protein | 3.15 |
|  | DT050252 | DT050252 | Putative uncharacterized protein | 3.11 |
|  | TC78977 | TC217721 | Putative uncharacterized protein | 3.11 |
|  | TC69240 | DR452655 | Putative uncharacterized protein | 3.10 |
|  | TC66604 | TC195507 | Putative uncharacterized protein | 3.10 |
|  | TC78713 | TC224770 | Putative uncharacterized protein | 3.07 |
|  | DV849907 | DN802280 | Putative uncharacterized protein | 3.07 |
|  | TC79584 | TC208598 | Pollen allergen Betv1 | 3.06 |
|  | CD486506 | CD486506 | Auxin-repressed protein | 3.06 |
|  | TC78628 | TC217527 | Wsv144 - White spot syndrome virus | 3.05 |
|  | TC62849 | TC201305 | Fb37 - Gossypium hirsutum | 3.04 |
|  | TC70366 | CO084207 | Histone deacetylase 2 | 3.04 |
|  | TC76312 | TC196485 | Putative uncharacterized protein | 3.03 |
|  | CO116628 | CO116628 | Putative uncharacterized protein | 3.03 |
| 0 DPA | TC78041 | DW233865 | Genome sequencing data contig C295 | 4.16 |
|  | TC78426 | TC184416 | Fibroin 1a | 3.80 |
|  | DT561813 | no hit | no data available | 3.47 |
|  | TC70356 | DT052928 | Putative uncharacterized protein | 3.43 |
|  | TC60141 | TC196696 | 4-coumarate:CoA ligase | 3.33 |
|  | TC79551 | TC215673 | Putative uncharacterized protein | 3.30 |
|  | DT459845 | DT459845 | Binding-protein-dependent transport systems inner membrane component precursor | 3.21 |
|  | TC69045 | TC211394 | Bax inhibitor-like protein | 3.01 |
|  | DR458893 | DR458893 | Putative uncharacterized protein | 2.94 |
|  | TC76566 | TC211651 | Putative uncharacterized protein | 2.86 |
|  | DN760847 | TC221410 | Putative uncharacterized protein | 2.86 |
|  | TC76964 | TC221766 | Putative uncharacterized protein | 2.85 |
|  | BF276279 | BF276279 | Expressed protein | 2.84 |
|  | TC60458 | TC209905 | NADH-ubiquinone oxidoreductase chain 1 | 2.80 |
|  | DT455623 | DT455623 | whole genome shotgun sequence | 2.75 |
|  | AI055589 | AI055589 | protein kinase-like protein | 2.74 |
|  | DT543487 | TC199707 | Putative uncharacterized protein | 2.74 |
|  | TC76248 | TC210246 | Amino acid carrier | 2.73 |
|  | TC68975 | TC226816 | Putative uncharacterized protein | 2.73 |
|  | DN827534 | DN827708 | Proline-rich protein-2 | 2.72 |
|  | TC79919 | TC192139 | Putative uncharacterized protein | 2.67 |
|  | TC62861 | TC213351 | Putative uncharacterized protein | 2.64 |
|  | TC77708 | DT050397 | LemA family protein precursor | 2.64 |
|  | TC71898 | DW500789 | *Oriza sativa* 01g0610400 protein | 2.63 |
|  | TC59522 | TC212143 | Microtubule associated protein | 2.63 |
|  | TC79128 | TC225592 | Protein STRUBBELIG-RECEPTOR FAMILY 3 precursor | 2.63 |
|  | TC62746 | TC183002 | Putative uncharacterized protein | 2.62 |
|  | TC62762 | TC205783 | Putative uncharacterized protein | 2.61 |
|  | TC60458 | TC209905 | NADH-ubiquinone oxidoreductase chain 1 | 2.59 |
|  | TC73768 | EX164235 | Cysteine protease | 2.58 |
|  | TC58960 | TC225941 | Elongation factor 1-alpha | 2.56 |
|  | TC63569 | TC212613 | Uncharacterized protein R871 | 2.55 |
|  | TC74829 | DW233552 | Putative uncharacterized protein | 2.49 |
|  | TC80304 | TC218198 | Putative uncharacterized protein | 2.48 |
|  | TC79184 | TC224959 | Putative uncharacterized protein | 2.48 |
|  | TC65253 | TC207185 | Putative uncharacterized protein | 2.47 |
|  | TC64557 | TC192876 | Putative uncharacterized protein | 2.47 |
|  | DR456332 | DR456332 | Putative uncharacterized protein | 2.46 |
|  | DR461858 | DR461858 | Ribonucleoside-diphosphate reductase small chain | 2.43 |
|  | TC65808 | ES829903 | hypothetical protein | 2.43 |
|  | TC80024 | ES793431 | CCR4-associated factor-like protein | 2.40 |
|  | TC75196 | TC222510 | Serine/threonine protein phosphatase | 2.40 |
|  | TC75492 | TC214448 | Enod93 protein | 2.40 |
|  | TC62633 | TC193562 | PIR | 2.39 |
|  | TC68097 | TC218113 | Putative uncharacterized protein | 2.38 |
|  | TC69756 | TC212541 | *Gossypium hirsutum* cDNA clone GH_CHX17K15 | 2.38 |
|  | TC76694 | TC205703 | Putative uncharacterized protein | 2.37 |
|  | TC79142 | No hit | No data available | 2.37 |
|  | TC67060 | TC188510 | Putative uncharacterized protein | 2.36 |
|  | TC70677 | TC212344 | Putative uncharacterized protein | 2.36 |
| 2 DPA | TC69642 | TC200576 | Putative uncharacterized protein | 3.32 |
|  | DN827534 | DN827708 | Proline-rich protein-2 | 3.24 |
|  | TC73538 | TC225337 | core eudicotyledons | 2.45 |
|  | TC70515 | TC185037 | Putative uncharacterized protein | 2.26 |
|  | TC78041 | DW233865 | Genome sequencing data contig C295 | 2.19 |
|  | TC60458 | TC209905 | NADH-ubiquinone oxidoreductase chain 1 | 2.19 |
|  | TC70302 | TC223007 | ATP synthase C chain | 2.18 |
|  | TC68759 | TC205313 | DnaJ-like protein | 2.16 |
|  | AI727497 | AI727497 | S-adenosylmethionine synthetase | 2.14 |
|  | BF272301 | BF272301 | calmodulin-1 | 2.13 |
|  | TC78984 | TC228871 | Env-like protein | 2.10 |
|  | TC58998 | DW233574 | Putative uncharacterized protein | 2.09 |
|  | TC75250 | TC186179 | NAM-like protein | 2.09 |
|  | BE052240 | BE052240 | S-adenosylmethionine synthetase | 2.05 |
|  | TC62067 | TC198349 | Putative uncharacterized protein | 2.04 |
|  | TC74141 | TC181539 | Dihydroflavonol 4-reductase | 2.03 |
|  | NP385858 | TC207561 | GHMYB36 | 2.02 |
|  | BG442282 | BG442282 | putative senescence-associated protein | 2.00 |
|  | TC60458 | TC209905 | NADH-ubiquinone oxidoreductase chain 1 | 2.00 |
|  | TC58926 | EV494209 | S-adenosylmethionine synthetase | 2.00 |
|  | TC59559 | BG441015 | Superoxide dismutase | 2.00 |
|  | TC73462 | EX172064 | Dihydroflavonol 4-reductase | 1.98 |
|  | TC75054 | TC189945 | Pol protein | 1.96 |
|  | DT458844 | ES847286 | Predicted protein | 1.95 |
|  | TC65994 | TC179685 | NAD(P)H-quinone oxidoreductase chain 4 | 1.94 |
|  | TC62607 | BE054331 | AER (*Nicotiana tabacum*) | 1.93 |
|  | TC60141 | TC196696 | 4-coumarate:CoA ligase | 1.92 |
|  | TC73914 | No hit | No data available | 1.89 |
|  | DT459198 | DT459198 | Putative uncharacterized protein | 1.88 |
|  | TC73256 | TC197179 | Putative uncharacterized protein | 1.88 |
|  | AY779338 | ES842269 | AP2/EREBP transcription factor ERF-2 | 1.87 |
|  | TC75684 | EV497280 | Chloroplast 30S ribosomal protein S3 | 1.86 |
|  | TC75026 | BF274727 | Adenosylhomocysteinase | 1.85 |
|  | TC67840 | TC208356 | Putative uncharacterized protein | 1.84 |
|  | BE051956 | BE051956 | Probable aquaporin PIP2-8 | 1.84 |
|  | DR463318 | DR463318 | unknown protein | 1.83 |
|  | DR463007 | DR463007 | MYB transcription factor MYB75 (*Glycine max*) | 1.83 |
|  | TC76713 | TC201708 | MYB-like DNA-binding domain protein | 1.82 |
|  | BG440546 | BG440546 | S-adenosylmethionine synthetase | 1.82 |
|  | TC66154 | TC216357 | Chalcone synthase | 1.81 |
|  | TC67965 | TC193521 | Aquaporin 2(*Samanea saman* -Rain tree) | 1.81 |
|  | TC67118 | TC213114 | Putative uncharacterized protein | 1.81 |
|  | BF276348 | TC203354 | hypothetical protein | 1.81 |
|  | TC79926 | TC219198 | Putative uncharacterized protein | 1.80 |
|  | TC72907 | DR459095 | Putative uncharacterized protein | 1.80 |
|  | TC65703 | TC197248 | Terminal flower 2 protein | 1.80 |
|  | TC66200 | ES796566 | Phenylalanine ammonia lyase | 1.80 |
|  | TC64813 | CF932161 | Putative uncharacterized protein | 1.79 |
|  | DT569461 | TC190220 | Predicted protein | 1.77 |
|  | TC67473 | TC211787 | ATP synthase protein 9 | 1.76 |
| 7 DPA | TC62928 | TC210651 | Putative uncharacterized protein | 3.50 |
|  | AI731676 | AI731676 | Medicago truncatula related | 3.06 |
|  | TC76310 | BE055698 | Sugar transferase | 2.44 |
|  | AI729534 | AI729534 | Embryo-specific protein 1 | 2.34 |
|  | TC67053 | TC179730 | Polygalacturonase-inhibiting protein precursor | 2.32 |
|  | TC75246 | EV492772 | Putative uncharacterized protein | 2.30 |
|  | CO108299 | CO108299 | Putative uncharacterized protein | 2.29 |
|  | DR454131 | TC225586 | Putative uncharacterized protein | 2.29 |
|  | TC60082 | DT052301 | Putative uncharacterized protein | 2.26 |
|  | TC61521 | TC185559 | Putative uncharacterized protein | 2.17 |
|  | BG439980 | BG439980 | MAP3K-like protein kinase | 2.17 |
|  | AI055325 | AI055325 | Putative uncharacterized protein | 2.16 |
|  | TC74338 | TC195952 | Putative uncharacterized protein | 2.15 |
|  | DT461550 | DT461550 | Putative uncharacterized protein | 2.14 |
|  | TC76600 | TC179646 | (+)-delta-cadinene synthase isozyme A | 2.13 |
|  | TC75077 | TC206580 | Putative uncharacterized protein | 2.13 |
|  | CO098023 | CO098023 | Putative uncharacterized protein | 2.13 |
|  | CO112055 | CO112055 | OJ991113_30.24 protein (*Oryza sativa*) | 2.10 |
|  | TC72819 | TC183183 | Cyclin-U2-1 | 2.09 |
|  | TC62223 | TC205772 | Fimbrin 2 | 2.07 |
|  | AY189970 | CO089081 | Profilin | 2.07 |
|  | TC75953 | DW008269 | MYB transcription factor (*Cucumis sativus*) | 2.05 |
|  | BG442977 | BG442977 | Jp18 (*Poncirus trifoliate*) | 2.03 |
|  | TC62638 | BG443726 | Putative uncharacterized protein | 2.02 |
|  | TC77261 | BG446532 | T12C24.5 (*Arabidopsis thaliana*) | 2.01 |
|  | TC70547 | TC225333 | Putative uncharacterized protein | 2.00 |
|  | AI725781 | AI725781 | SPX N-terminal | 2.00 |
|  | TC59091 | TC184219 | Beta-ketoacyl-CoA synthase | 2.00 |
|  | TC80112 | EX170820 | whole genome shotgun sequence | 1.99 |
|  | TC71402 | TC221889 | Putative uncharacterized protein | 1.99 |
|  | TC68075 | AI729781 | Putative uncharacterized protein | 1.99 |
|  | AW187888 | AW187888 | Putative uncharacterized protein | 1.97 |
|  | TC60726 | EV489897 | Putative uncharacterized protein | 1.96 |
|  | AW186957 | AW186957 | Putative uncharacterized protein | 1.96 |
|  | TC68965 | BQ407434 | Putative uncharacterized protein | 1.96 |
|  | TC76211 | TC192354 | Putative uncharacterized protein | 1.95 |
|  | TC60096 | ES848070 | Putative uncharacterized protein | 1.95 |
|  | AW587498 | AW587498 | Fimbrin 2 | 1.95 |
|  | TC69055 | CO495717 | Putative uncharacterized protein | 1.95 |
|  | BG440195 | BG440195 | Putative uncharacterized protein | 1.94 |
|  | BG443011 | BM358666 | whole genome shotgun sequence | 1.94 |
|  | TC74692 | BE052063 | At4g34750 (*Arabidopsis thaliana*) | 1.93 |
|  | TC77032 | TC206666 | Carboxyl-terminal proteinase-like (*Oryza sativa*) | 1.93 |
|  | DT571813 | TC183199 | Phi-1 protein (*Nicotiana tabacum*) | 1.93 |
|  | TC67087 | TC225015 | Galactinol synthase(*Brassica napus*) | 1.92 |
|  | AI730776 | AI730776 | Receptor protein kinase-like protein | 1.92 |
|  | TC78654 | TC209930 | Putative uncharacterized protein | 1.92 |
|  | TC72086 | BQ403722 | Putative uncharacterized protein | 1.92 |
|  | AI726575 | AI726575 | At3g26700 (*Arabidopsis thaliana*) | 1.92 |
|  | TC62588 | TC194787 | Putative uncharacterized protein | 1.91 |
