# Supplementary material for: Activation of Arabidopsis Seed Hair Development by Cotton Fiber-Related Genes
Source: PLoS One. 2011 Jul 11;6(7):e21301. doi: 10.1371/journal.pone.0021301 (PMC3136922; doi:10.1371/journal.pone.0021301)
Supplement: Table S5 — A list of representative genes in each cluster. (DOC) [file pone.0021301.s005.doc]

**Table S5.** A list of representative genes in each cluster

| Cluster | EST ID_CGI8 | EST ID_CGI10 | Tentative annotation |
| --- | --- | --- | --- |
| 1 | AI727497 | AI727497 | SAM1 (S-adenosylmethionine synthetase 1) |
|  | BE052240 | BE052240 | SAM-2 (S-adenosylmethionine synthetase 2) |
|  | EV494209 | TC58926 | SAM-2 (S-adenosylmethionine synthetase 2) |
|  | CO123850 | TC60696 | pyruvate kinase, putative |
|  | TC185774 | TC69088 | Mitogen-activated protein kinase |
|  | TC188276 | TC70118 | Cis-prenyltransferase |
|  | TC221684 | TC71501 | Ser/Thr protein kinase |
|  | TC186532 | TC72046 | adenylylsulfate kinase, putative |
| 2 | AI055589 | AI055589 | serine/threonine protein kinase, putative |
|  | AI725469 | AI725469 | Shaggy-related protein kinase zeta |
|  | CO094001 | CO094001 | ribitol kinase, putative |
|  | TC186857 | DT543487 | Pollen-specific kinase partner protein |
|  | EX169169 | TC73047 | Protein E6 |
|  | TC63543 | TC229085 | Putative pumilio/Mpt5 family RNA-binding protein |
|  | TC228380 | TC76694 | Phosphatidylinositol 4-kinase |
|  | TC201582 | TC77576 | leucine-rich repeat transmembrane protein kinase, putative |
| 3 | BM359206 | BM359206 | Zinc finger CCCH domain-containing protein ZFN-like |
|  | TC184928 | TC59978 | Zinc finger A20 and AN1 domain-containing stress-associated protein 1 |
|  | ES851548 | TC78190 | Calcineurin-like phosphoesterase-like protein |
|  | TC211558 | TC77847 | Acid phosphatase 1 precursor |
|  | CO084207 | TC70366 | Histone deacetylase 2 |
|  | TC184811 | TC73631 | Beta-galactosidase - Gossypium hirsutum |
|  | TC212040 | TC76555 | FAC1 (EMBRYONIC FACTOR1) |
| 4 | TC196854 | TC60063 | Gibberellin induced protein |
|  | BQ407450 | TC60196 | ribonuclease activity |
|  | TC217467 | TC73239 | Nuclear receptor coactivator 6 |
|  | ES846728 | TC73445 | 60S ribosomal protein L8 (RPL8C) |
|  | TC189378 | TC75387 | PS6 (RIBOSOMAL PROTEIN S6) |
|  | TC210802 | TC64286 | Ribosomal-like protein |
|  | TC190173 | TC68777 | 50S ribosomal protein L24 |
| 5 | TC196424 | TC63972 | Zinc finger protein-like |
|  | TC196347 | TC67191 | Heat shock transcription factor 29 |
|  | TC190658 | TC74741 | ABF2 (Abscisic acid responsive elements-binding factor2) |
|  | ES810346 | TC75739 | MYB6 |
|  | TC196508 | TC76014 | transcription factor |
| 6 | TC63385 | TC63385 | ATSWI3C (Arabidopsis thaliana switching protein 3C) |
|  | TC191841 | TC66508 | auxin/aluminum-responsive protein, putative |
|  | TC66510 | TC66510 | auxin/aluminum-responsive protein, putative |
|  | TC186034 | TC66798 | ATP binding / damaged DNA binding |
|  | TC191948 | TC67889 | Homeobox-leucine zipper protein HAT22 |
|  | TC229448 | TC69040 | ATSC35 (arginine/serine-rich splicing factor 35) |
|  | EV486345 | TC69095 | BZIP transcription factor bZIP124 |
|  | DT458844 | DT458844 | Auxin-responsive protein IAA20 (Indoleacetic acid-induced protein 20) |
|  | DW505454 | TC65219 | gibberellin-regulated family protein |
|  | TC79957 | TC79957 | gibberellin-regulated family protein |
