# Supplementary material for: Activation of Arabidopsis Seed Hair Development by Cotton Fiber-Related Genes
Source: PLoS One. 2011 Jul 11;6(7):e21301. doi: 10.1371/journal.pone.0021301 (PMC3136922; doi:10.1371/journal.pone.0021301)
Supplement: Table S6 — Primer sequences used for RT-PCR and quantitative RT-PCR analyses. (DOC) [file pone.0021301.s006.doc]

**Table S6.** Primer sequences used for RT-PCR and quantitative RT-PCR analyses

| EST ID_CGI8 | EST ID_CGI10 | Gene Name | Quantitative RT-PCR Primers used |
| --- | --- | --- | --- |
| AI730621 | AI730621 | Ring zinc finger proterin | F: CTT TTC TGC TGG CCT TGC TT |
|  |  |  | R:GCA GAC AGT GTG AAA TTC CCG |
| TC62849 | TC201305 | Fb37 | F:CCC ACC TCT TTG AAA CTC TTG GCT |
|  |  |  | R: CAA TTG TTC GCA CAC AGC TTC GGT |
| TC74600 | TC183307 | Transcription factor | F:GTG ATG ATG TTA CCA GCC CCA |
|  |  |  | R:AGC ACC AGT TGC GGA TAC ACA |
| TC75739 | TC229318 | GhMyb112b | F: AAC CGC AAT GCC TCG ATG TAC TCA |
|  |  |  | R:ACG TCG ATG TTG GAG ATT GGA GGT |
| AY072821 | AY072821 | GhRDL | F:ATC AGC ATG GAA CCC TAA GCA |
|  |  |  | R:GGA CCC AAA CAA TGT GAT CCC |
| TC75912 | TC187769 | ABC transporter | F:CCA TGG CTG CAA TGG ATT AAG |
|  |  |  | R:CCA AGT GCC CAA AAA CAT CTG |
| TC58926 | TC208063 | SAM-2 | F: GGC AGC AAC ATC TTT GCT GAT GGA |
|  |  |  | R: AAG CAA TGC TGC TTT CAC CAC CTG |
| TC60696 | CO123850 | pyruvate kinase | F: TAA GCC TGC GAG CTT CGG TTC TAA |
|  |  |  | R: GGA ATA ATT TCA GGT TCG GCG CGT |
| TC69088 | TC185774 | ATMPK2 | F: GCA CTA GCA ATG CCA AGG GTC AAT |
|  |  |  | R: TTG TCA CAG CAG AGG AGA AGC TCA |
| CO094001 | CO094001 | ribitol kinase | F: GAC ACC ATG CAG GTC ATT CAT CCA |
|  |  |  | R: GCG ACA GCT GCT GCT GAT AAA GTT |
| DT543487 | TC199707 | ROPGEF1 | F: TTC AGC ACA CTC CTT ATG CCT CTC |
|  |  |  | R: TCT CTT CAA AGA GGA AGG TGC CCA |
| TC73047 | EX169169 | SRPK4 | F: ATG GAG GCA ATG GAG GTT GCA ATG |
|  |  |  | R: TGG AAG AAG AAA TGG CTG CGT TGG |
| AF024716 | AF024716 | HISTONE3 | F: GAA GCC TCA TCG ATA CCG TC |
|  |  |  | R: CTA CCA CTA CCA TCA TGG C |
| AY072821 | AY072821 | GhRDL1-R | R: TTT GGC AGC TTA TAG CTC CA |
|  |  | GFP:GhRDL1 | F: CCA TAG TGA CTG GAT ATG TT |
|  |  | GhMYB2:YFP | R: TCT ACC TTC CCA CAA TTC GT |
|  |  | 35S | F: GAT CTC TCT GCC GAC AGT GG |
|  |  | BETA-6 TUBULIN | F: ACC ACT CCT AGC TTT GGT GAT C |
|  |  |  | R: AGG TTC ACT GCG AGC TTC CTC A |
| AY072821 |  | cDNA cloning | F:CGCGGATCCGCGGGGAATTAGTCACTCCTGTTCTAG (*Bam*H1) |
|  |  |  | R: ATAAGAATGCGGCCGCTAAACTATTTACTTAGG GACCCAAACAATGTGA (*NotI*) |
| F: forward primer, R: reverse primer | | | |
