# Supplementary figures and images for: Activation of Arabidopsis Seed Hair Development by Cotton Fiber-Related Genes
Source: PLoS One. 2011 Jul 11;6(7):e21301. doi: 10.1371/journal.pone.0021301 (PMC3136922; doi:10.1371/journal.pone.0021301)

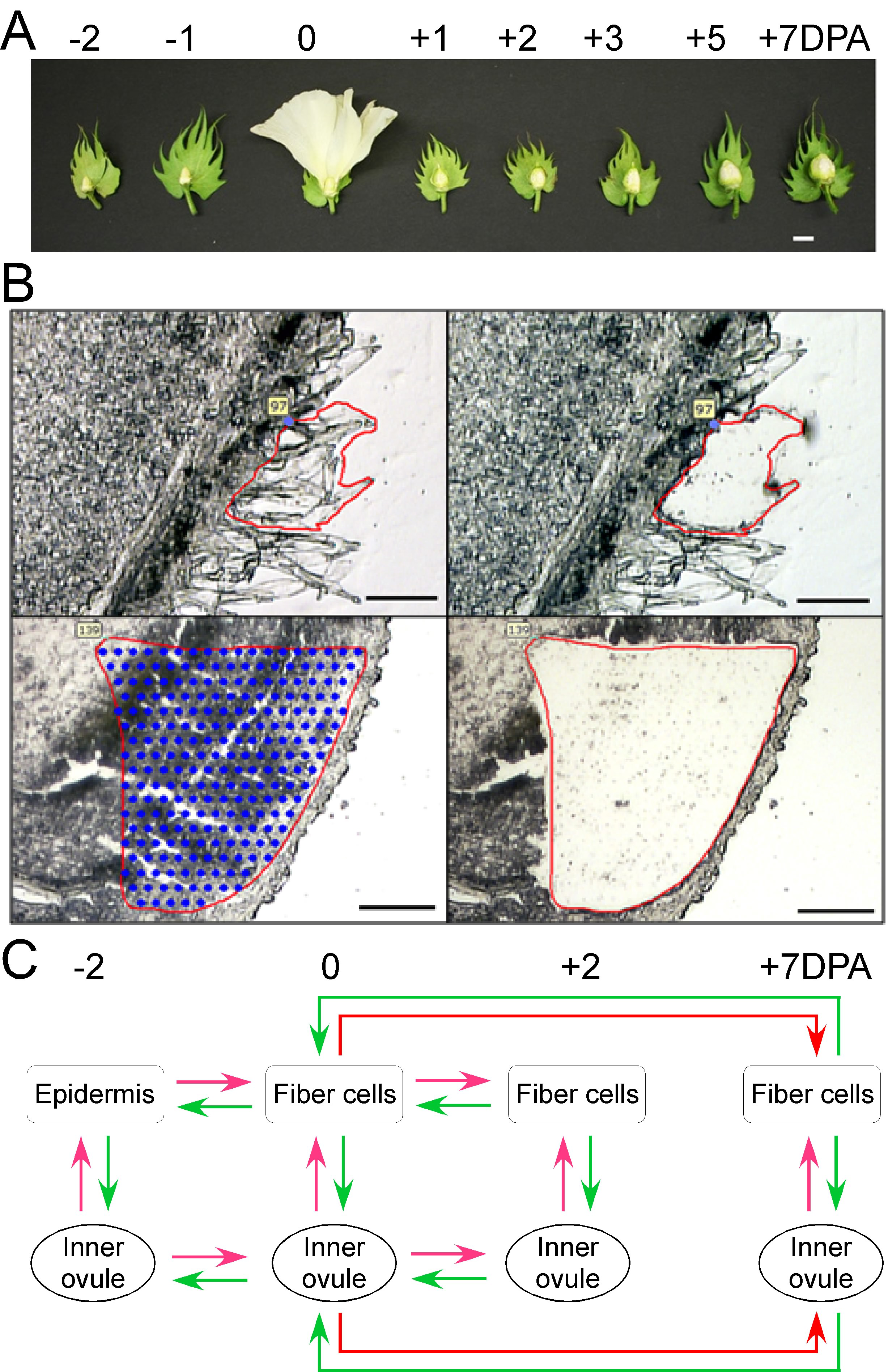

Supplement: Figure S1 — Microarray experimental design and application of laser capture microdissection (LCM). (A) Development of cotton fiber. (B) Application of LCM in fibers (2 DPA, upper panel) and inner integuments of ovules (2 DPA, lower panel). The captured cells were removed (right photos in each panel). (C) Microarray experimental design. Arrows denote dye swaps used in each hybridization (red: Cy5, green: Cy3). (TIF) [file pone.0021301.s007.tif]

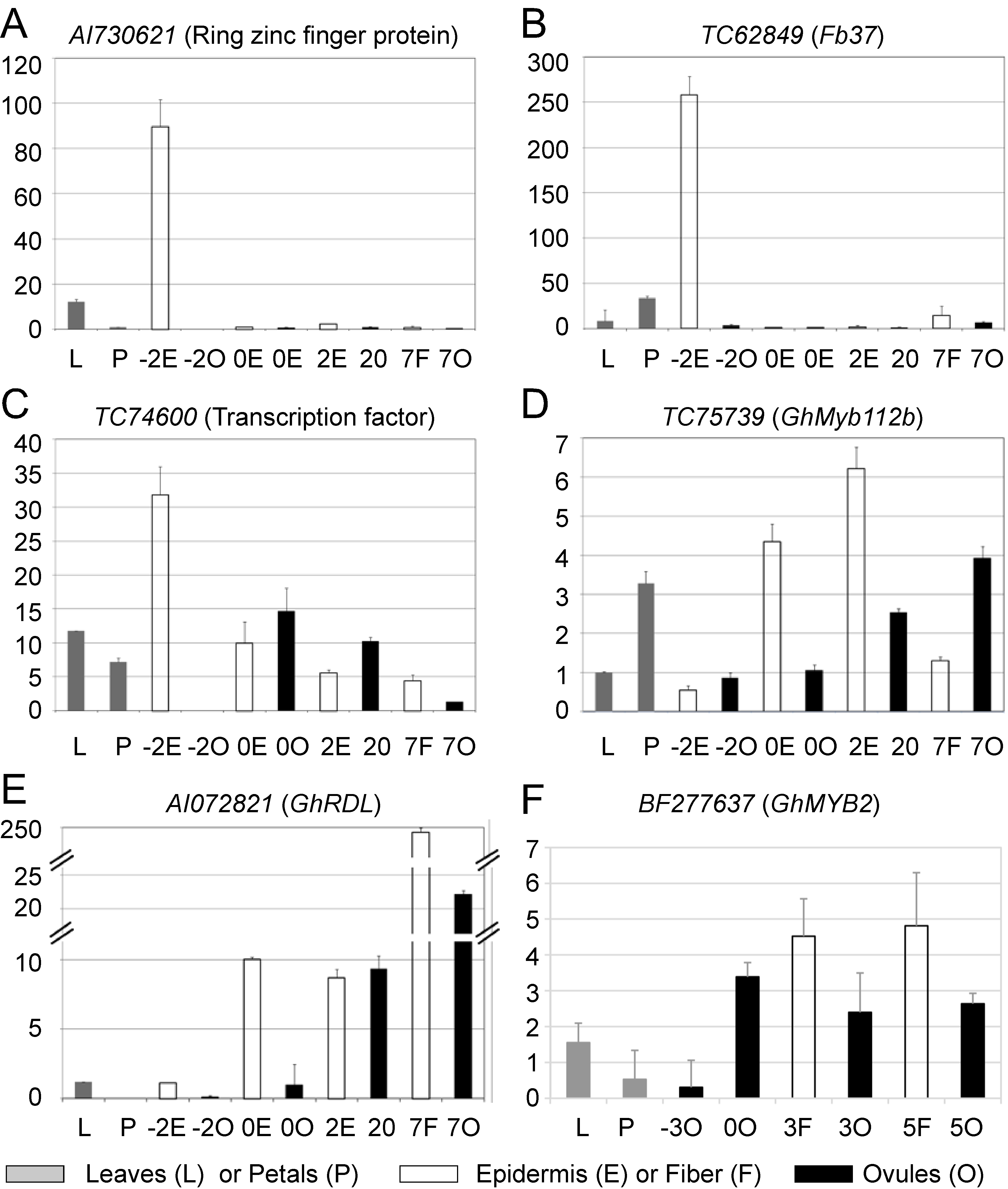

Supplement: Figure S2 — qRT-PCR validation of six differentially expressed genes detected by microarrays. Gene expression was analyzed in ten tissues, namely, leaves (L), petals (P), protodermal cells (−2E), fiber cell initials (0E, 0 DPA and 2E, 2 DPA), fibers (7F, 7 DPA), ovules at −2 DPA (−2O), 0 DPA (0O), 2 DPA (2O), and 7 DPA (7O), respectively. (A) Ring zinc finger protein (AI730621). (B) Fiber protein 37 (TC62849). (C) Transcription factor (TC74600). (D) GhMYB112b (TC75739). (E) GhRDL (AY072821). (F) ABC transporter (TC75912). (TIF) [file pone.0021301.s008.tif]

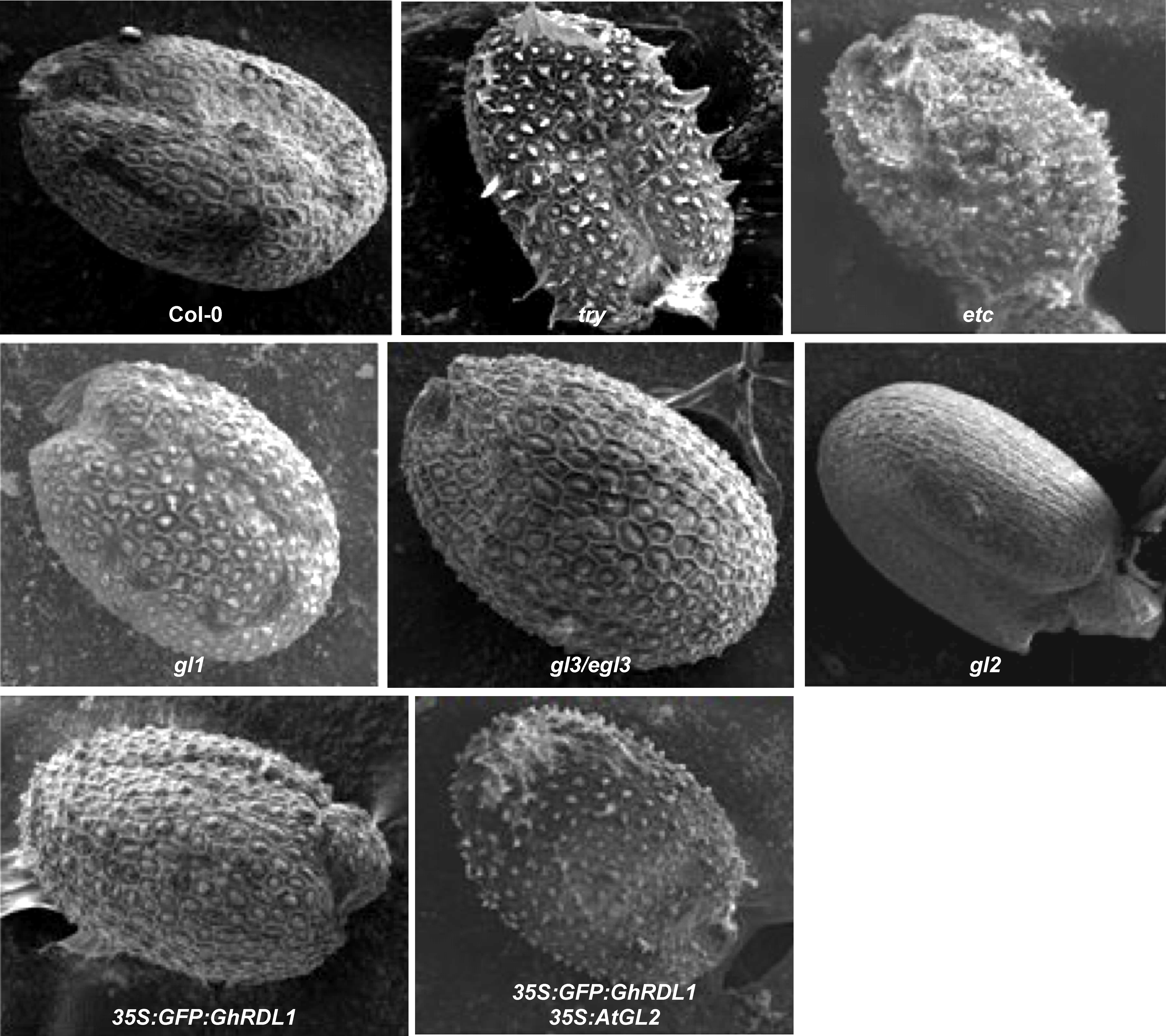

Supplement: Figure S4 — Effects of trichome regulatory genes on mucilage formation and columella cell morphology. Col-0: A. thaliana Col-0 shows relatively smooth seed surface. Hair-like structure was observed in try, etc, 35S:GFP:GhRDL1 and 35S:GFP:GhRDL1x35S:AtGL2 seeds. The seed surface was rough, but no obvious hair-like structure was observed in gl1 and gl3/egl3 seeds. The seed surface of gl2 seed was extremely smooth, suggesting loss of mucilage and columella cells. (TIF) [file pone.0021301.s010.tif]
