# Supplementary material for: Activation of Arabidopsis Seed Hair Development by Cotton Fiber-Related Genes
Source: PLoS One. 2011 Jul 11;6(7):e21301. doi: 10.1371/journal.pone.0021301 (PMC3136922; doi:10.1371/journal.pone.0021301)
Supplement: Figure S3 — Sequence alignment of REPOSNSE TO DESSICATION 22 (RD22) and RD22-like ( RDL ) genes. GenBank accession numbers are AAL67991 (Gossypium hirsutum RDL1, GhRDL1), AAT66912 (Gossypium arboreum RDL1, GaRDL), and Q08298 (Arabidopsis thaliana RD22, AtRD22). (DOC) [file pone.0021301.s009.doc]

GhRDL MKVLSPILACL-ALAVVVSHAALSPEQYWSYKLPNTPMPKAVKEILHPELMEEKSTSVNV 59

GaRDL1 MKVLSPILACL-ALAVVVSHAALSPEQYWSYKLPNTPMPKAVKEILHPELMEEKSTSVNV 59

AtRD22 MAIRLPLICLLGSFMVVAIAADLTPERYWSTALPNTPIPNSLHNLLTFDFTDEKSTNVQV 60

GhRDL GGGGVNVN----------------------TGKGKPGGDTHVNVG-----GKGVGVNTGK 92

GaRDL1 GGGGVNVN----------------------TGKGKPGGDTHVNVG-----GKGVGVNTGK 92

AtRD22 GKGGVNVNTHKGKTGSGTAVNVGKGGVRVDTGKGKPGGGTHVSVGSGKGHGGGVAVHTGK 120

GhRDL PG---------GGTHVNV--------------GDPFNYLYAASETQIHEDPNVALFFLEK 129

GaRDL1 PG---------GGTHVND--------------PDPFNYLYAASETQIHEDPNVALFFLEK 129

AtRD22 PGKRTDVGVGKGGVTVHTRHKGRPIYVGVKPGANPFVYNYAAKETQLHDDPNAALFFLEK 180

GhRDL DMHPGATMSLHFTENT---EKSAFLPYQTAQKIPFSSDKLPEIFNKFSVKPGSLKAEMMK 186

GaRDL1 DMHPGATMSLHFIENT---EKSAFLPYQTAPKNTFSSDKLPEIFNKFSVKPGSVKAEMMK 186

AtRD22 DLVRGKEMNVRFNAEDGYGGKTAFLPRGEAETVPFGSEKFSETLKRFSVEAGSEEAEMMK 240

GhRDL NTIKECEQPAIEGEEKYCATSLESMIDYSISKLGKVDQ-AVSTEVEKQ-TPMQKYTIAAG 244

GaRDL1 NTIKECEQPAIEGEEKYCATSLESMIDYSISKLGKVDQ-AVSTEVEKQ-TPMQKYTIAAG 244

AtRD22 KTIEECEARKVSGEEKYCATSLESMVDFSVSKLGKYHVRAVSTEVAKKNAPMQKYKIAAA 300

GhRDL -VQKMTDDKAVVCHKQNYAYAVFYCHKSETTRAYMVPLEGADGTKAKAVAVCHTDTSAWN 303

GaRDL1 -VQKMTDDKAVVCHKQNYAYAVFYCHKSETTRAYMVPLEGAGGTKAKALAVCHTDTSAWN 303

AtRD22 GVKKLSDDKSVVCHKQKYPFAVFYCHKAMMTTVYAVPLEGENGMRAKAVAVCHKNTSAWN 360

GhRDL PKHLAFQVLKVEPGTIPVCHFLPRDHIVWVPK 335

GaRDL1 PKHLAFQFLKVEPGTIPVCHFLPRDHIVWVPK 335

AtRD22 PNHLAFKVLKVKPGTVPVCHFLPETHVVWFSY 392

**Figure S3. Sequence alignment of RESPONSE TO DESSICATION 22 (RD22) and RD22-like (*RDL*) genes.** GenBank accession numbers are AAL67991 (*Gossypium hirsutum* RDL1, GhRDL1), AAT66912 (*Gossypium arboreum* RDL1, GaRDL), and Q08298 (*Arabidopsis thaliana* RD22, AtRD22).
